# Neuronal Responses to Ischemia: Scoping Review of Insights from Human-Derived In Vitro Models

**DOI:** 10.1007/s10571-023-01368-y

**Published:** 2023-06-28

**Authors:** Eva J. H. F. Voogd, Monica Frega, Jeannette Hofmeijer

**Affiliations:** 1 Clinical Neurophysiology, University of Twente, Enschede, The Netherlands; 2 Department of Neurology, Rijnstate Hospital, Arnhem, The Netherlands

**Keywords:** Cerebral ischemia, Human in vitro models, SH-SY5Y, HiPSC-derived neurons, Cell death, Apoptosis

## Abstract

Translation of neuroprotective treatment effects from experimental animal models to patients with cerebral ischemia has been challenging. Since pathophysiological processes may vary across species, an experimental model to clarify human-specific neuronal pathomechanisms may help. We conducted a scoping review of the literature on human neuronal in vitro models that have been used to study neuronal responses to ischemia or hypoxia, the parts of the pathophysiological cascade that have been investigated in those models, and evidence on effects of interventions. We included 147 studies on four different human neuronal models. The majority of the studies (132/147) was conducted in SH-SY5Y cells, which is a cancerous cell line derived from a single neuroblastoma patient. Of these, 119/132 used undifferentiated SH-SY5Y cells, that lack many neuronal characteristics. Two studies used healthy human induced pluripotent stem cell derived neuronal networks. Most studies used microscopic measures and established hypoxia induced cell death, oxidative stress, or inflammation. Only one study investigated the effect of hypoxia on neuronal network functionality using micro-electrode arrays. Treatment targets included oxidative stress, inflammation, cell death, and neuronal network stimulation. We discuss (dis)advantages of the various model systems and propose future perspectives for research into human neuronal responses to ischemia or hypoxia.

**Graphical Abstract:**

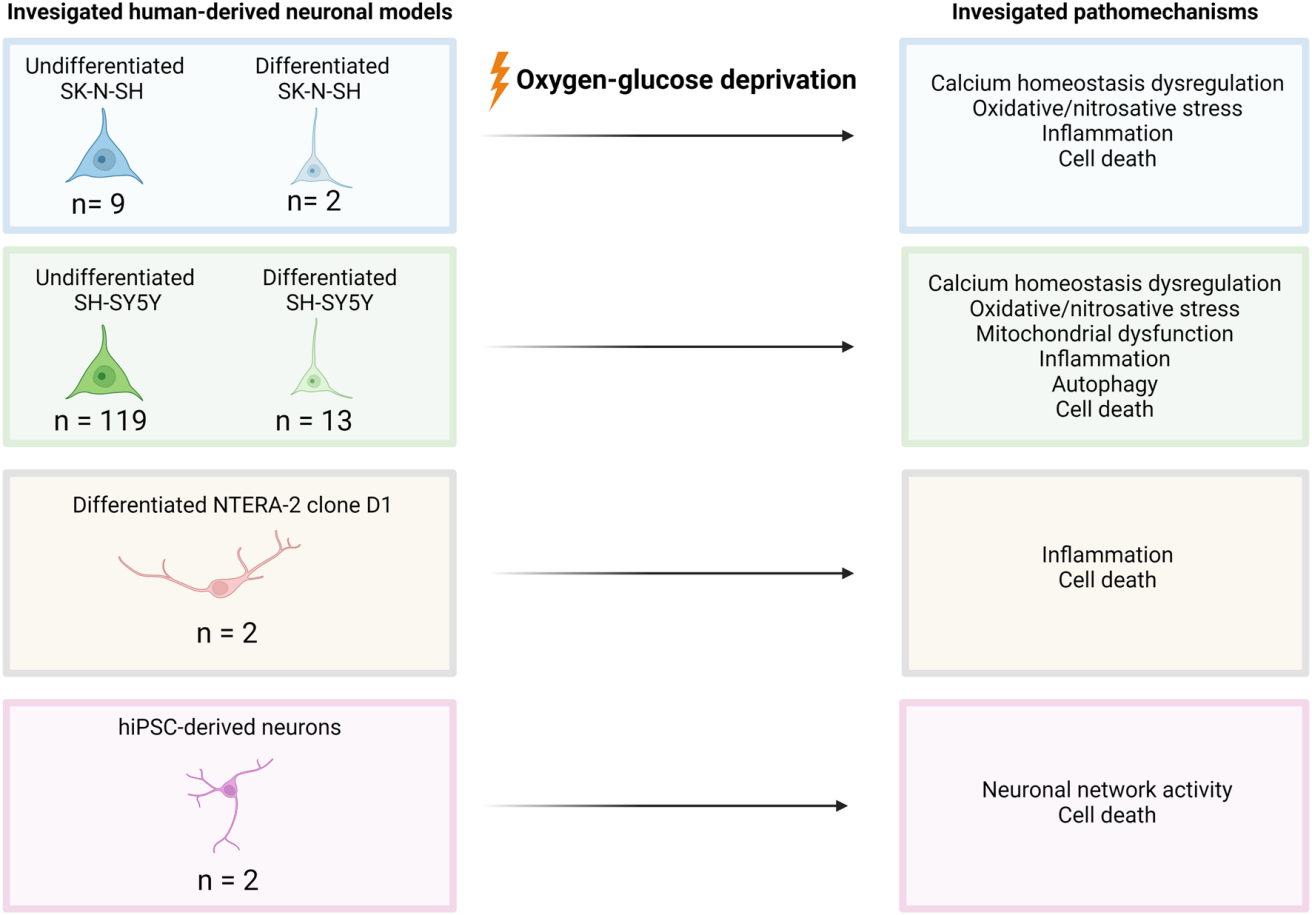

**Supplementary Information:**

The online version contains supplementary material available at 10.1007/s10571-023-01368-y.

## Introduction

It has been challenging to translate neuroprotective treatment effects from experimental animal models to patients with cerebral ischemia. While numerous rodent studies have shown efficacy of divergent “neuroprotective” treatment strategies, in clinical trials these treatments have failed to improve functional outcomes (Ginsberg 2008). Failure of treatments in clinical trials have been discussed extensively, with possible explanations ranging from methodological weaknesses of animal studies to differences in treatment protocols and from infarct sizes to patient selection (Ginsberg 2008). However, genetic differences between rodents and humans that may translate to neurophysiological differences are relatively undiscussed. While rodents and humans share part of their DNA, various genes and proteins are expressed differently (Mestas and Hughes 2004). Therefore, cellular responses to cerebral ischemia or hypoxia may differ between rodents and humans. Furthermore, mechanisms of action or pathways that are targeted with neuroprotective treatments can vary (Ginsberg 2008). Consequently, rodent models might not be the optimal starting point to investigate the effect of cerebral ischemia on neuronal functionality and treatment strategies.

Various experimental studies addressed responses to ischemia or hypoxia in human-derived cell models. These consist of non-neuronal and neuronal models. The most used human in vitro model to study neuronal responses to ischemia or hypoxia is the neuroblastoma-derived SH-SY5Y cell model (Liu et al. 2018). This cell model is based on a cancerous cell line with the corresponding genetic characteristics, which may affect responses to ischemia or hypoxia and treatment strategies (Biedler et al. 1973, 1978). SH-SY5Y cells can, in principle, be differentiated into neuron-like cells upon stimulation (Shipley et al. 2016). However, the majority of research with SH-SY5Y cell models is conducted in undifferentiated SH-SY5Y cells. Only a small proportion of the research made use of protocols to differentiate SH-SY5Y cells into neuron-like cells. Furthermore, most studies focused on cell viability, neglecting neuronal functionality (Liu et al. 2018).

The recent advancement of human induced pluripotent stem cell (hiPSC) technology has created new opportunities to establish human neuronal in vitro models and investigate responses to ischemia or hypoxia. HiPSCs can be derived from healthy donors or patients, capturing person-specific genetic characteristics, and differentiated into neurons to generate neuronal networks (Frega et al. 2017; Mossink et al. 2021a). This allows investigation of the effect of, for example, a genetic mutation on neuronal functionality (Mossink et al. 2021b).

With this scoping review of the literature, we aim to provide an overview of (1) human neuronal in vitro models that have been used to study neuronal responses to ischemia or hypoxia, including characteristics of the various models, (2) parts of the ischemic pathophysiological cascade that have been investigated and (3) treatment targets that have been established in those human neuronal models. We will use the results as a starting point to discuss advantages and disadvantages of the various model systems, highlight current knowledge gaps, and propose possible future perspectives for research into human neuronal responses to ischemia or hypoxia.

## Methods

For this scoping review, we followed the PRISMA guidelines with regard to literature search, data collection, presentation of study characteristics and results, and discussion. However, we did not include a quality appraisal or risk of bias analysis.

To review investigated human neuronal in vitro models of cerebral ischemia or hypoxia, we applied a search in PubMed and SCOPUS databases until November 2021. We conducted the literature research with several combinations of key words and MeSH terms. We searched the literature with general terms for “human neuronal in vitro models” and the following specific search terms: “NT2-N”, “SK-N-SH”, “SH-SY5Y” or “hiPSC-derived neurons” (Liu et al. 2018). For selection of disease we used the MeSH terms “ischemic stroke” or “cerebral ischemia” and search terms “hypoxia” or “oxygen–glucose deprivation (OGD)” in combination with the different human neuronal in vitro models. One reviewer (EV) screened articles for eligibility based on the abstracts and methods. Review articles were used to screen reference lists. We only included studies with modeling of cerebral ischemia by oxygen–glucose deprivation or hypoxia. Studies were excluded when chemical simulation of ischemia was investigated. Flow chart is provided in the supplementary materials. For analyses of effects of potential neuroprotective treatments, we included studies on treatments that were applied during or after ischemia/hypoxia, and excluded studies that investigated the effect of pre-treatment (i.e. a treatment started before initiation of hypoxia or ischemia). We extracted the cell type, the level of differentiation towards neurons (if applicable), the way in which ischemia or hypoxia was modelled (including duration and recovery period), treatment strategy (if applicable), outcome measures, and results from the included studies. All results of the scoping review are presented in a descriptive way.

## Results

We included 147 papers on four different human neuronal in vitro models of cerebral ischemia/hypoxia.

### Model Characteristics

**Fig. 1:**
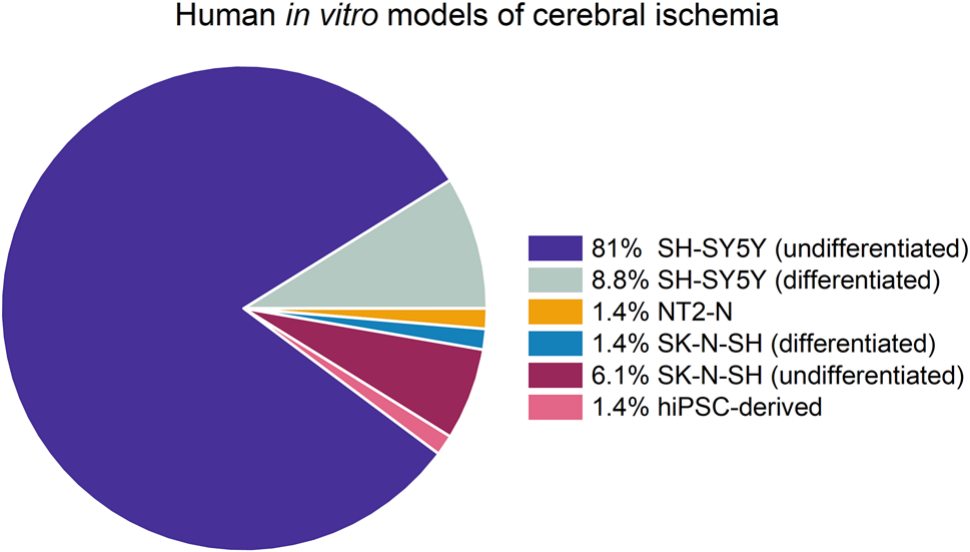
Pie chart representing the distribution of human neuronal in vitro models used in experimental cerebral ischemia studies. The majority of the research is conducted in SH-SY5Y cell models. *hiPSCs* human induced pluripotent stem cells. Differentiated NT2-N = teratocarcinoma-derived Ntera2/D1 neuron-like cells

**Fig. 2:**
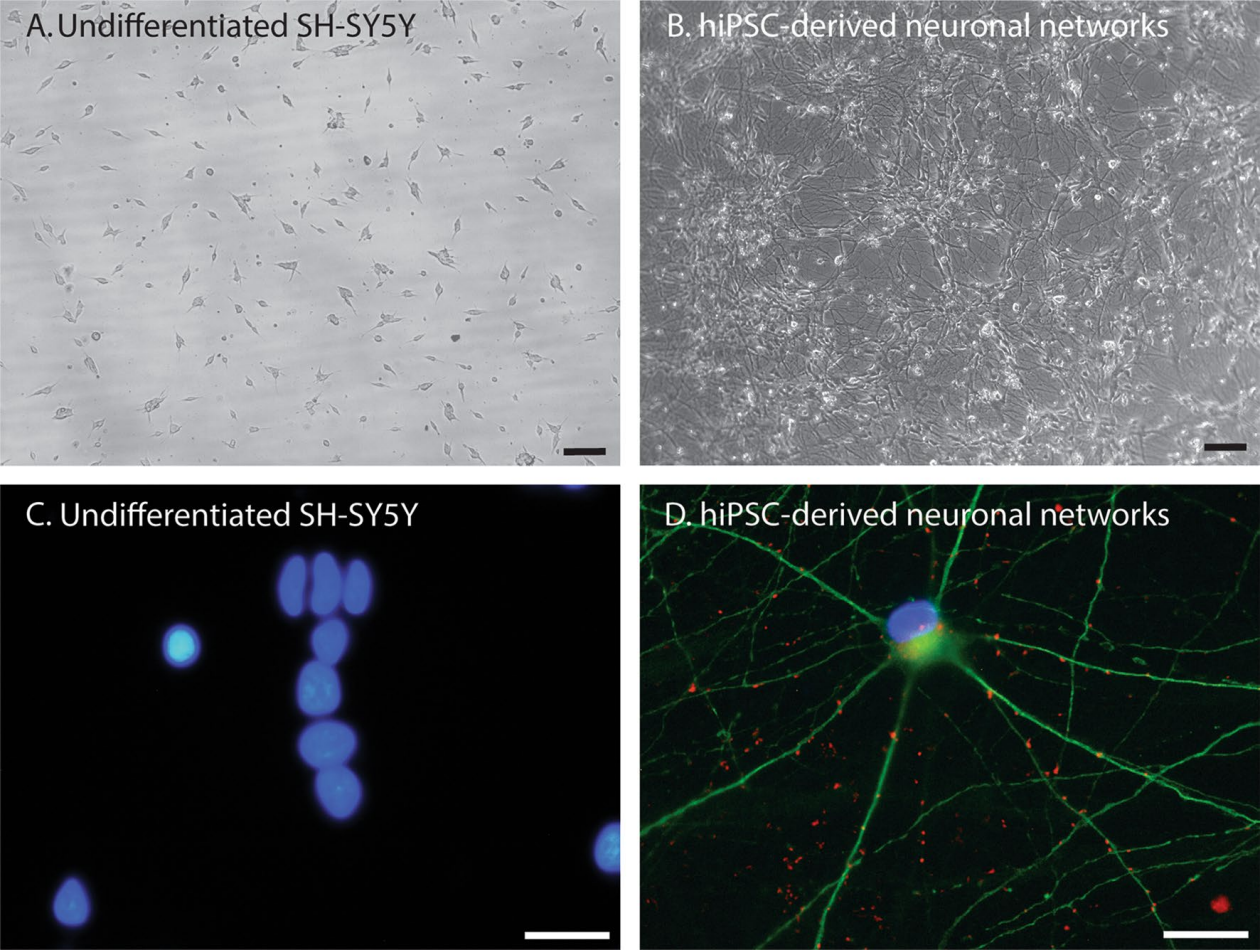
Representative images of undifferentiated SH-SY5Y cells and hiPSC-derived neuronal networks. Bright field images of undifferentiated SH-SY5Y cells (**A**) and hiPSC-derived neuronal networks (**B**) (× 4 magnification) (scalebar = 100 μm). Immunofluorescent images of undifferentiated SH-SY5Y cells (**C**) and hiPSC-derived neuronal networks (**D**) (× 60 magnification) (scalebar = 30 μm). Undifferentiated SH-SY5Y cells have short truncated processes and do not express the neuronal markers MAP2 and Synapsin 1/2 positive puncta. HiPSC-derived neuronal networks have extended neurites and express neuronal markers MAP2 and Synapsin 1/2 positive puncta. Blue = DAPI (nuclei), Green = micro-tubule associated protein 2 (MAP2), Red = Synapsin 1/2

Model characteristics are summarized Fig. 1 and Table 1. Of the 147 included papers, 145 were on cell models derived from unhealthy donors (neuroblastoma SK-N-SH (n = 11) and SH-SY5Y (n = 132)) or carcinoma (differentiated NT2-N (n = 2)). SK-N-SH is a cell model directly derived from a neuroblastoma cell line. SH-SY5Y cell model is based on a subclone derived from the SK-N-SH cell line. These can both be differentiated into neuron-like cells by stimulation with retinoic acid (RA). However, the majority of studies on neuronal response to ischemia/hypoxia is conducted in undifferentiated cell models (SK-N-SH undifferentiated n = 9 and differentiated n = 2; SH-SY5Y undifferentiated n = 119, and differentiated n = 13). Two studies were conducted on neurons differentiated from hiPSCs of healthy donors (Juntunen et al. 2020; Pires Monteiro et al. 2021). Examples of cultured undifferentiated SH-SY5Y and hiPSC-derived neuronal networks are presented in Fig. 2, showing that undifferentiated SH-SY5Y cells have short truncated processes and do not express the neuronal markers microtubule-associated protein 2 (MAP2) and Synapsin 1/2 positive synaptic puncta. While hiPSC-derived neuronal networks show long neurites and express MAP2 and Synapsin 1/2 positive synaptic puncta.

**Table 1:** Overview of human-derived neuronal cell models investigated in experimental ischemia

Cell model	Cell type	Differentiation	Type of experimental ischemia	Duration	Recovery	Methods	Results
SH-SY5Y	Neuroblastoma	Yes [1–14]	OGD	40 min to 30 h	0–72 h	LDH-assay MTT-assay CCK-8 assay IF Western blot RT-PCR	↑ Cell membrane injury ↓ Cell survival ↑ Cell death ↑ Pro-apoptotic factors ↓ Anti-apoptotic factors ↑ Pro-inflammatory factors ↓ Anti-inflammatory factors ↑ ROS signaling ↑ Axonal damage
		No [15–134]	OGD Hypoxia	30 min to 72 h	0–72 h	LDH-assay MTT-assay CCK-8 assay IF Western blot RT-PCR	↑ Cell membrane injury ↓ Cell survival ↑ Pro-inflammatory factors ↓ Anti-inflammatory factors ↑ Mitochondrial dysfunction ↑ Cell death ↑ Pro-apoptotic factors ↓ Anti-apoptotic factors ↑ ROS signaling ↓ Calcium regulation ↑ Autophagy
SK-N-SH	Neuroblastoma	Yes [135, 136]	OGD Hypoxia	8 h	15 h	LDH-assay Western blot IF Annexin V-FITC/PI RT-PCR Atomical iron levels Chromatin immunoprecipitation assay	↑ Intracellular iron uptake ↑ Iron-dependent cell death ↑ Cell membrane injury ↑ Cell membrane injury ↑ KIF2 ↑ ROS signaling ↑ Cell death ↑ Pro-apoptotic factors ↑ Pro-inflammatory factors
		No [121, 137, 144]	OGD Hypoxia	12–24 h	0–72 h	MTT LDH-assay CCK-8 ELISA RT-qPCR Luciferase reporter assay Annexin V-FITC/PI Western blot Metabolic labeling IF	↑ Cell membrane injury ↓ Cell survival ↑ PTEN expression ↑ Cell death ↑ Pro-apoptotic factors ↑ Cell membrane injury ↓ Intracellular ATP content ↑ Increased cell swelling ↑ Pro-inflammatory factors
NT2-N	Embryonic carcinoma	Yes [145, 146]	OGD Hypoxia	3 h	21 h	CELISA IF	↓ Cell survival ↓ Expression of complement system factors
HiPSC-derived neurons	Healthy cell lines derived from fibroblasts	Yes [7, 147]	OGD Hypoxia	6–48 h	24–72 h	IF CyQuant analysis RT-PCR Electrophysiological activity Live/Dead assay incl. apoptosis	↓ Cell survival ↓ Proliferation ↑ Axonal damage ↓ Spontaneous firing activity ↓ Synchronous events of neuronal networks ↓ Cell survival ↑ Apoptosis ↑ Cell death

Reference list for this table is provided in the supplementary materials

*OGD* oxygen glucose deprivation; *LDH* lactate dehydrogenase; *MTT* 3-(4,5-dimethylthiazol-2-yl)-2,5-diphenyltetrazolium bromide; *CCK-8* cell counting kit 8; *IF* immunofluorescence; *RT-PCR* reverse transcription polymerase chain reaction; *ELISA* enzyme-linked immunosorbent assay; *CELISA* cellular ELISA; ROS reactive oxygen species; *PTEN* phosphatase and tensin homolog; *ATP* adenosine triphosphate

Cerebral ischemia was modeled by oxygen–glucose deprivation (OGD; n = 142) or hypoxia (n = 5). All studies used immunocytochemical techniques to investigate cell survival, protein expression linked with cell death or inflammation, or factors related to oxidative stress. Neuronal functionality was assessed in only one study in which electrophysiological measurements were performed with micro-electrode arrays (MEAs) (Pires Monteiro et al. 2021).

### Effects of Ischemia or Hypoxia on Human Cell Models

The pathophysiological cascade that follows upon cerebral ischemia/hypoxia is extensive and complex. In SH-SY5Y, SK-N-SH, NT2-N and hiPSC-derived cell models, several steps of this pathophysiological cascade have been investigated. These steps are summarized in Table 2 and explained below.

**Table 2:** Overview of pathomechanisms investigated in human-derived neuronal cell models of experimental ischemia

Pathology	Factors	Undifferentiated SH-SY5Y	Differentiated SH-SY5Y	Undifferentiated SK-N-SH	Differentiated SK-N-SH	hiPSC-derived neurons	NT2-N
Calcium homeostasis dysregulation	Ca^2+^ extruders	Na^+^/Ca^2+^ exchanger 1 [34]					
Oxidative stress	Oxidant	Reactive oxygen species Superoxide anion [72] Malondialdehyde [29, 35, 36, 100, 102, 107, 113] peroxynitrites [72] NOX [43, 44, 110] Nitrite [44] hypoxia inducible factor 1 [37]		Malondialdehyde [136]			
	Anti-oxidant	Superoxide dismutase [29, 33, 35, 36, 58, 65, 102, 107, 109, 113] Glutathione [37, 100, 107, 136] NADPH [37] Catalase [102] NF-E2 related factor 2 [62, 65, 79, 83, 89, 107, 132] NQO1 [65, 79, 83, 89, 107, 132] HO-1 [65, 79, 83, 89, 107, 132] neuronal NO synthase [93]					
	Other factors	GRP78/BIP [43, 70, 100] p-IRE1α [43, 70, 100] XBP1 [43, 70, 100] CHOP [43, 70, 100] ATF6 [70] TGF-β/SMAD [118] APE1 [110]	ERMP1 [4, 9] GRP78 [4, 9] p-PERK [4, 9] p-EIF2α [4, 9] CHOP [4, 9]				
Mitochondrial dysfunction		Cyclophilin D [113] mitochondrial membrane depolarization [15, 79, 103] MMP [63, 79, 102] Nuclear respiratory factor-1 [61] Mitochondrial transcription factor A [61] Mitophagy [80]	MMP [8, 10]				
Inflammation	Pro-inflammatory	IL-1β [17, 29, 56, 58, 64, 87, 88, 121, 130] IL-6 [47, 56–58, 64, 75, 87, 88, 130] IL-18 [121] IFN-γ [130] NLRP3 [121] TNF [17, 29, 40, 47, 54, 56–58, 64, 75, 87, 88, 117, 130, 132] FASL [21, 125] FBXO3 [58] NF-κB [40, 47, 48, 50, 58, 82, 87, 96, 117, 132] DUSP14 [87] TLR4 [23, 47] MyD88 [23, 47] SIRT6 [130] SIRT1 [130] HMGB1 [55, 132] CXCL1 [88] IκB [29, 47, 50, 58]	NLRP3 [5]	IL-1β [121] IL-10 [121]			CD55 [146]
	Anti-inflammatory	MAFB [126] IL-10 [29]					CD59 [145]
Autophagy		LC3 [53, 64, 68, 78, 85, 101, 106, 132, 133] CX32 [80] NUR77 [80] Beclin-1 [85, 101, 106, 132] DUSP5 [101] mTOR [32, 36, 39, 58, 64, 65, 73, 92, 96, 102, 109, 110, 120, 132, 133]	LC3 [2, 13] Beclin-1 [2] mTOR [2]				
Apoptosis	Pro-apoptotic	Cell membrane injury [18, 24, 36, 44, 57, 71, 73, 77, 79, 83, 102, 103, 106, 107, 110–112, 127, 131, 132] BIK [24] BAX [16, 28, 33, 36, 38, 41, 45, 46, 58, 59, 67,69, 98, 100, 104, 106, 110, 113, 115, 125, 130, 133] cyt-c [45, 59, 61, 102] BIM [41, 92] BCL2L14 [119] Caspase-3,-9 [16,20, 21, 24, 28, 30, 33, 36, 43, 46, 49, 57, 61, 67, 73, 79, 83, 84, 87, 92, 94, 97, 98, 100, 102, 105, 109, 110, 112, 113, 115, 120–123, 125, 130, 132, 134] P53 [131] AKT [32, 36, 39, 58, 64, 65, 73, 92, 96, 102, 109, 110, 120, 132, 133] PI3K [32, 36, 39, 58, 64, 65, 92, 96, 102, 109, 110, 120, 132, 133] FOXO [32, 36, 39, 58, 64, 65, 73, 92, 96, 102, 109, 110, 120, 132, 133] PTEN [36, 38, 39, 120, 137] DAPK1 [33] HSP20 [70] MMP-9 [59] MIF [59] FLT3 [28] TLR8 [95] MAPK/JNK/ERK [41, 63, 73, 82, 87, 96, 101, 104, 106, 117] IRF-1 [69] PDCD4 [84] ING5 [124]	Cell membrane injury [2–4, 8, 10–13] BAX [10] AIF [10] PARP [6, 10, 11] Caspase-3, -6, -7 [3, 10, 11, 13, 14] AKT [2, 6] GSK- 3β [8] MAPK/JNK/ERK [3, 4, 11, 12]	Cell membrane injury [135, 138, 141] PARP [138] Caspase-1,-3, -7 [121, 138, 144] PTEN [137	Caspase-3 [136]	Caspase-3/7 [147]	
	Anti-apoptotic	BCL-2 [20, 28, 33, 36, 38, 41, 45, 46, 58, 59, 67, 69, 97, 98, 104, 106, 108, 110, 113, 115, 125, 130, 132, 133] BCL-XL [16, 41, 59, 98] EGFR [111] Sphingosine kinase 2 [26] BDNF [20, 31, 104]	BCL-2 [10] BCL-W [11] EPCR [10] EGFR [8]				
Parthanatos		AIF [59, 100] PARP [59,83, 87, 100, 112]					
Cell death		Cell death rate [16, 18, 20, 22, 24, 28, 31, 33–36, 38, 39, 43–49, 51, 52, 54, 58, 60, 62, 65–68, 70–75, 77–79, 81, 83, 86–88, 91–96, 99, 102–113, 115–118, 120–122, 124–128, 131–134]	Cell death rate [2, 3, 5, 7, 9–11, 13]	Cell death rate [121, 135, 137]			

Reference list for this table is provided in the supplementary materials

*NADPH* Nicotinamide adenine dinucleotide phosphate; *NQO1* NAD(P)H dehydrogenase [quinone] 1; *HO-1* hemeoxygenase-1; *GRP78/BIP* 78 kDa glucose-regulated protein/binding immunoglobulin protein; *p-IRE1* phosphorylated inositol-requiring enzyme-1; *XBP1* X-box binding protein 1; *CHOP* C/EBP homologous protein; *ATF6* activating transcription factor 6; *TGF /SMAD* transforming growth factor/SMAD; *APE1* Apurinic/apyrimidinic endonuclease 1; *ERMP1* Endoplasmic Reticulum Metallopeptidase 1; *GRP78* 78 kDa glucose-regulated protein; *p-PERK* phosphorylated protein kinase-like endoplasmic reticulum kinase; *p-EIF2α* phosphorylated α subunit of eukaryotic initiation factor 2; *MMP* mitochondrial membrane potential; *IL-1β* interleukin-1β; *IL-6* interleukin-6; *IL-18* interleukin-18; *IFN-γ* interferon γ; *NLRP3* NLR family pyrin domain containing 3; *TNF* Tumor necrosis factor; *FASL* Fas ligand; *FBXO3* F-Box Protein 3; *NF-κB* Nuclear factor kappa-light-chain-enhancer of activated B cells; *DUSP14* Dual Specificity Phosphatase 14; *TLR4* Toll-like receptor 4; *MyD88* Myeloid differentiation primary response 88; *SIRT1* SIRTUIN 1; SIRT6 SIRTUIN 6; *HMGB1* High mobility group box 1; *CXCL1* chemokine (C-X-C motif) ligand 1; *IκB* Inhibitor of κB; *CD55* Complement decay-accelerating factor; *MAFB* V-maf musculoaponeurotic fibrosarcoma oncogene homolog B; *CD59* CD59 glycoprotein; *LC3* Microtubule-associated proteins 1A/1B light chain 3B; *CX32* connexin32; *NUR77* nuclear receptor 4A1; *DUSP5* Dual specificity protein phosphatase 5; *mTOR* mammalian target of rapamycin; *BAX* BCL2 associated X; *cyt-c* cytochrome-c; *AIF* apoptosis inducing factor; *PARP* poly(ADP-ribose) polymerase; *BIM* Bcl-2-like protein 11; *BCL2L14* Apoptosis facilitator Bcl-2-like protein 14; *P53* Tumor protein P53; *AKT* Protein kinase B; *PI3K* Phosphoinositide 3-kinases; *FOXO* forkhead box proteins; *PTEN* Phosphatase and tensin homolog; *DAPK1* Death-associated protein kinase 1; *HSP20* heat shock protein20; *MMP-9* matrix metallopeptidase 9; *MIF* macrophage migration inhibitory factor; *FLT3* FMS-like tyrosine kinase-3; *TLR8* Toll-like receptor 8; *IRF-1* Interferon regulatory factor-1; *PDCD4* ‘programmed cell death 4′; *ING5* Inhibitor of growth protein 5; *GSK- 3β* Glycogen synthase kinase 3β; *BCL-2* B-cell lymphoma 2; *BCL-XL* B-cell lymphoma-extra-large; *EGFR* epidermal growth factor receptor; *BDNF* brain derived neurotrophic factor; *BCL-W* Bcl-2-like protein 2; *EPCR* Endothelial protein C receptor

#### SH-SY5Y and SK-N-SH Cell Models

##### Calcium Homeostasis Dysregulation

Ischemia induced membrane depolarization leads to opening of calcium channels, ultimately resulting in an increase in intracellular Ca^2+^. With insufficient ATP production, Ca^2+^ extruders (e.g. Na^+^/Ca^2+^ exchanger) stop working, causing a pathological increase of the intracellular and mitochondrial Ca^2+^ concentration. The Na^+^/Ca^2+^ exchanger 1 (NCX1) has been investigated in one study using undifferentiated SH-SY5Y cells. The results showed that NCX1 was repressed during OGD by the RE1-silencing transcription factor (REST) (Formisano et al. 2013). In undifferentiated SK-N-SH cells increased levels of intracellular calcium was found during OGD (n = 1) (Lehane et al. 2013).

##### Oxidative/Nitrosative Stress

Oxidative stress results from imbalance between pro- and anti-oxidants, that in turn results in excessive formation of reactive oxygen species (ROS). Various negative effects of ROS on DNA, proteins or lipids have been established in SH-SY5Y and SK-N-SH cell models of cerebral ischemia.

The first ROS that is produced during ischemia/hypoxia is superoxide anion (O_2_^−^), which is the precursor of most other ROS. One study in undifferentiated SH-SY5Y cells found an increase in O_2_^−^ (Marmol et al. 2021). Superoxide dismutases (SODs) convert superoxide to hydrogen peroxide (H_2_O_2_), which can be removed by glutathione peroxidase (GPx). GPx was decreased after ischemia/hypoxia in undifferentiated SH-SY5Y and SK-N-SH cells (Wang et al. 2020a; Wang and Xu 2021). SODs prevent the formation of highly aggressive ROS. Ten studies in undifferentiated SH-SY5Y cells and one in undifferentiated SK-N-SH cells found decreased levels of SODs during ischemia/hypoxia (Di et al. 2021; Dong et al. 2021; Feng et al. 2021; Gao et al. 2017, 2020; Li et al. 2019b; Liu et al. 2019b; Wang et al. 2019b, 2020a; Wang and Xu 2021; Wen et al. 2020; Xu et al. 2020b).

Glutathione (GSH) is an antioxidant that can prevent damage to cellular components by ROS. The ratio of reduced GSH to oxidized glutathione (GSSG) is a measure of cellular oxidative stress. This ratio has been investigated in one study using undifferentiated SH-SY5Y cells (Guo et al. 2009). The results showed a decreased ratio by increased oxidized glutathione. This study also investigated the level of cellular nicotinamide adenine dinucleotide phosphate (NADPH) content. NADPH provides oxidation–reduction which protects against ROS allowing the regeneration of GSH. There was no difference in NADPH content between normoxia or hypoxia treated undifferentiated SH-SY5Y cells (Guo et al. 2009). NADPH oxidase was involved in post-ischemic cell death in differentiated SH-SY5Y cells (n = 1) (Beske and Jackson 2012). One other study investigated the level of GSH in undifferentiated SH-SY5Y cells and found that there was a decrease in GSH during hypoxia, leading to increased cellular oxidative stress (Wang et al. 2018a). Another anti-oxidant that has been investigated in undifferentiated SH-SY5Y cells is catalase. This enzyme was reduced after ischemia/hypoxia (Wang et al. 2019b).

Besides anti-oxidants, free radical scavengers were investigated in an undifferentiated SH-SY5Y cell model of cerebral ischemia, revealing that spermine, spermidine and other polyamines were elevated (Shin et al. 2016). ROS can also be decreased by hypoxia inducible factor 1 (HIF-1). In undifferentiated SH-SY5Y cells it was found that HIF-1 regulated redox status after hypoxic exposure (Guo et al. 2009).

Another factor that counteracts ROS formation is the activation of the redox sensitive transcription factor NF-E2 related factor 2 (NRF-2). This is a master regulator of enzymes involved in anti-oxidant glutathione synthesis and in elimination of ROS. Thus, NRF-2 plays a role in the defense against oxidative stress. NRF-2 and other cytoprotective enzymes have been investigated in seven studies using undifferentiated SH-SY5Y cells. The results showed increased expression of NRF-2 (Lin-Holderer et al. 2016; Liu et al. 2019b; Park et al. 2017; Ruan et al. 2020; Shi et al. 2019; Wang and Xu 2021; Zhi et al. 2020) and the cytoprotective enzymes NAD(P)H dehydrogenase [quinone] 1 (NQO1) and hemeoxygenase-1 (HO-1) (Liu et al. 2019b; Park et al. 2017; Ruan et al. 2020; Shi et al. 2019; Wang and Xu 2021; Zhi et al. 2020) after ischemia/hypoxia.

Malondialdehyde (MDA) is a marker of oxidative stress. It is a highly cytotoxic agent and has been investigated to assess oxidative stress in seven studies using undifferentiated SH-SY5Y cells and one study using undifferentiated SK-N-SH cells. MDA levels increased during hypoxia/ischemia, which suggested increased oxidative stress (Dong et al. 2021; Gao et al. 2017, 2020; Wang et al. 2018a, 2019b, 2020a; Wang and Xu 2021; Xu et al. 2020b). Nicotinamide adenine dinucleotide phosphate oxidase (NOX) is an important player in ROS generation. NOX expression was increased in undifferentiated SH-SY5Y cell models of cerebral ischemia (n = 3) (Hong et al. 2017; Hsieh et al. 2021; Wu et al. 2020a).

The neuronal NO synthase (nNOS) enzymes are expressed in neurons and contribute to nitrite oxide (NO) production in ischemic processes, leading to nitrosative stress. nNOS has been investigated in one study using undifferentiated SH-SY5Y cells and the results showed reduced expression of nNOS enzymes during ischemic stress (Tajes et al. 2013). Nitrosative stress is mediated by peroxynitrites, that are produced by NO. Peroxynitrites play an important role in the development of neuroinflammation. One study found increased peroxynitrite levels after hypoxia in undifferentiated SH-SY5Y cells (Marmol et al. 2021). The level of nitrite has been investigated in undifferentiated SH-SY5Y cells and the results showed increased intracellular nitrite concentration after ischemia/hypoxia (Hsieh et al. 2021).

ROS regulates cell ferroptosis by multiple signaling pathways like the Janus kinase and signal transducer and activator of transcription (JAK/STAT). JAK2 has been investigated in undifferentiated SH-SY5Y cells in OGD. The results showed increased expression of JAK2 and STAT3 (n = 2) (Huang et al. 2018; Xu et al. 2020b).

Oxidative stress can lead to endoplasmic reticulum (ER) stress-related protein expression. ER stress markers are inducers of neuronal cell death and increase the tissue injury after cerebral ischemia. In three studies with undifferentiated SH-SY5Y cells, several ER stress related proteins were increased, such as 78 kDa glucose-regulated protein/binding immunoglobulin protein (GRP78/BIP), phosphorylated inositol-requiring enzyme-1 alpha (p-IRE1α), X-box binding protein 1 (XBP1), C/EBP homologous protein (CHOP), and activating transcription factor 6 (ATF6) (Hong et al. 2017; Lu et al. 2019; Wang et al. 2018a). In two studies with differentiated SH-SY5Y cells, Endoplasmic Reticulum Metallopeptidase 1 (ERMP1), GRP78, phosphorylated protein kinase-like endoplasmic reticulum kinase (p-PERK), phosphorylated α-subunit of eukaryotic initiation factor 2 (p-EIF2α) and CHOP were increased (Chi et al. 2019; Pan et al. 2020).

The transforming growth factor (TGF)/SMAD pathway has been implicated in oxidative stress. TGF expression was increased in an undifferentiated SH-SY5Y cell model of cerebral ischemia, together with increased phosphorylation of SMAD2 and SMAD3. Ischemia/hypoxia promoted translocation of SMAD3 from the cytoplasm to the nucleus (Yang et al. 2021b).

Oxidative stress can cause DNA damage. A multifunctional protein that is involved in the base-excision repair of oxidative DNA damage is Apurinic/apyrimidinic endonuclease 1 (APE1). The expression level of APE1 decreased during ischemia/hypoxia in undifferentiated SH-SY5Y cells (n = 1) (Wu et al. 2020a).

##### Mitochondrial Dysfunction

Neuronal ischemia/hypoxia causes mitochondrial dysfunction along several ways: oxidative stress with mitochondrial DNA mutations, dysfunction of the mitochondrial respiratory chain, altered membrane permeability with Ca^2+^ accumulation, and disturbance of mitochondrial defense systems (Ham and Raju 2017).

One study in undifferentiated SH-SY5Y cells showed that the mitochondrial permeability transition pore is opened with OGD by translocation of P53 to mitochondria and activation of Cyclophilin D (Zhao et al. 2013). The consequent mitochondrial membrane depolarization has been found in undifferentiated SH-SY5Y cells (n = 3) (Agudo-Lopez et al. 2010; Park et al. 2017; Wang et al. 2018b). Mitochondrial membrane permeabilization by opening of the mitochondrial permeability transition pore and loss of the mitochondrial membrane potential (MMP) has also been established in three studies in undifferentiated and in two studies differentiated SH-SY5Y cells (Lin et al. 2020; Liu et al. 2019a; Park et al. 2017; Sriwastva et al. 2020; Wang et al. 2019b). Normal mitochondrial function was reduced in undifferentiated SH-SY5Y cells after OGD (Herrmann et al. 2013).

Nuclear respiratory factor-1 and mitochondrial transcription factor A were decreased in undifferentiated SH-SY5Y cells after ischemia/hypoxia (Lin et al. 2020). Cytochrome oxidase activity was decreased (Lin et al. 2020). When mitochondrial dysfunction occurs, cells usually remove the dysfunctional mitochondria with mitophagy. Ischemia/hypoxia induced mitophagy has been established in one study in undifferentiated SH-SY5Y cells (Ping et al. 2021).

##### Inflammation

Cerebral ischemic injury and reperfusion cause a detrimental inflammatory cascade. Cytokines play a crucial role. Several cytokines have been investigated in human neuronal in vitro models of cerebral ischemia. These cytokines can be divided in pro-inflammatory and anti-inflammatory cytokines. In undifferentiated SH-SY5Y (n = 12) and SK-N-SH (n = 1) cell models, expression of pro-inflammatory cytokines (interleukin-1β, interleukin-6, interleukin-18 and interferon γ) was increased after ischemia/hypoxia (Chai et al. 2020; Dong et al. 2021; Huang et al. 2021; Li et al. 2019a, b; Li and Ma 2020; Liu et al. 2021; Meng et al. 2021; Shi et al. 2020, 2021; Yin et al. 2021; Zhao and Wang 2020). Pro-inflammatory cytokines like IL-1β and IL-18 can be activated by NLR family pyrin domain containing 3 (NLRP3) inflammasomes, which comprises NLRP3, ASC, and pro-caspase-1. The formation of the NLRP3 inflammasome was increased in undifferentiated SH-SY5Y cells (n = 1), differentiated SH-SY5Y (n = 1) and undifferentiated SK-N-SH cells (Fu et al. 2020; Yin et al. 2021).

Anti-inflammatory cytokines can be induced by V-maf musculoaponeurotic fibrosarcoma oncogene homolog B. This transcriptional activator was found to be decreased after ischemia/hypoxia in one study using undifferentiated SH-SY5Y cells (Zhang et al. 2020a). The anti-inflammatory factor interleukin-10 was reduced after ischemia/hypoxia in one study with undifferentiated SH-SY5Y cells (Dong et al. 2021).

Tumor necrosis factor (TNF) is released by cells under stress and stimulates the immune response. The expression of TNF is only investigated in undifferentiated SH-SY5Y cells during and after ischemia/hypoxia (n = 15). The results show an increase in TNF expression induced by ischemia/hypoxia, which suggests increased inflammatory response (Chai et al. 2020; Dong et al. 2021; Hao et al. 2015; Huang et al. 2021; Landgraf et al. 2020; Li et al. 2019a; Li and Ma 2020; Liu et al. 2021; Meng et al. 2021; Shi et al. 2020, 2021; Yang et al. 2014; Zhao and Wang 2020; Zhi et al. 2020). Another protein that belongs to the TNF family is Fas ligand (FASL). When this protein binds to its receptor, it induces apoptosis. FASL has been investigated in two studies using undifferentiated SH-SY5Y cells and the results showed Fas/FASL interaction was increased. This was correlated with a higher apoptosis rate (Chen et al. 2015; Zhang et al. 2016b). Another study has found that the levels of TNF and interleukin-6 (IL-6) were regulated by F-Box Protein 3 (FBXO3). Ischemia/hypoxia induced increased levels of FBXO3, which was correlated with increased levels of TNF and IL-6 (Li et al. 2019b). Various pro-inflammatory factors (nuclear factor kappa-light-chain-enhancer of activated B cells (NF-κB), Dual Specificity Phosphatase 14 (DUSP14), Toll-like receptor 4 (TLR4), Myeloid differentiation primary response 88 (MYD88), SIRTUIN 1 (SIRT1), SIRTUIN 6 (SIRT6), High mobility group box 1 (HMGB1), chemokine (C-X-C motif) ligand 1 (CXCL1)) were increased in 14 studies with undifferentiated SH-SY5Y cells (Chen et al. 2021a; Dong et al. 2021; Hao et al. 2015; Huang et al. 2021; Janssen et al. 2021; Jiang et al. 2012; Lee et al. 2013; Li et al. 2019b; Ruan et al. 2021; Shi et al. 2020, 2021; Tian et al. 2020; Yang et al. 2014; Zhao and Wang 2020; Zhi et al. 2020).

Inhibitor of κB (IκB) kinase is an enzyme involved in propagating the cellular response to inflammation. It activates NF-κB. This kinase is investigated in four studies using undifferentiated SH-SY5Y cell, two of which investigated IκB kinase in combination with the activity of NF-κB (Dong et al. 2021; Huang et al. 2021; Jiang et al. 2012; Li et al. 2019b). The results showed an increase in IκB kinase.

##### Autophagy

Intracellular autophagy is activated in different cell types of the brain upon nutrient starvation or metabolic stress. It is the phagocytosis of cytoplasmic materials, which are captured by autophagosomes and fuse with lysosomes to form autolysosomes and are then degraded. Several factors related to autophagy after ischemia/hypoxia have been investigated in human neuronal in vitro models.

Ischemia/hypoxia increased the level of microtubule-associated protein 1 light chain 3 (LC3), as was found in undifferentiated (n = 9) (Lai et al. 2020; Liu et al. 2016, 2021; Niu et al. 2018; Shi et al. 2012; Wang et al. 2017, 2019c; Zhi et al. 2020; Zhou et al. 2019) and differentiated SH-SY5Y cells (n = 2) (Cheng et al. 2019; Zhang et al. 2019b). Connexin32 (CX32) was found to inhibit the autophagic effect of nuclear receptor 4A1 (NUR77) during ischemia/hypoxia in one study using undifferentiated SH-SY5Y cells (Ping et al. 2021). Increased Beclin-1 expression was found to increase the level of autophagy in four studies using undifferentiated SH-SY5Y cells (Shi et al. 2012; Wang et al. 2017, 2019c; Zhi et al. 2020) and one study using differentiated SH-SY5Y cells during ischemia/hypoxia (Cheng et al. 2019).

Dual specificity protein phosphatase 5 (DUSP5) increased the level of autophagy through DUSP5-ERK1/2 axis according to one study in undifferentiated SH-SY5Y cells (Wang et al. 2017). Phosphorylation of mammalian target of rapamycin (mTOR) was found to be increased, which was correlated with increased level of autophagy in five studies using undifferentiated SH-SY5Y cells (Guo et al. 2018; Liu et al. 2021; Wang et al. 2019b; Zhi et al. 2020; Zhou et al. 2019) and one study using differentiated SH-SY5Y cells (Cheng et al. 2019).

##### Cell Death

Cell death is a much studied final common path in human neuronal in vitro models of cerebral ischemia. A total of 59 studies in undifferentiated (Agudo-Lopez et al. 2010; Castri et al. 2014; Chen et al. 2019; Dong et al. 2019, 2021; Du et al. 2012; Feng et al. 2021; Gao et al. 2017, 2020; He et al. 2014; Hu et al. 2020; Huang et al. 2018, 2021; Jia et al. 2021; Lee et al. 2013; Li and Ma 2020; Li et al. 2020a; Liu et al. 2019b; Lu et al. 2019; Luan et al. 2013; Marutani et al. 2012; Meng et al. 2021; Nampoothiri and Rajanikant 2019; Niu et al. 2018; Park et al. 2017; Ruan et al. 2020; Shan et al. 2022; Shi et al. 2021; Song et al. 2019; Tian et al. 2020; Wang et al. 2013, 2017, 2018b, c, 2019b, c, 2020b, 2021a, b; Wang and Xu 2021; Wen et al. 2020; Wu et al. 2020a, b; Xing et al. 2021; Xu et al. 2020a, b; Yan et al. 2015, 2020; Yang et al. 2014, 2021a; Yao et al. 2019; Yi et al. 2020; Zappala et al. 2021; Zhang et al. 2016a, b, 2019a, b, 2020; Zhao and Wang 2020; Zhao et al. 2013; Zhi et al. 2020; Zhou et al. 2019; Zuo et al. 2020) and four studies in differentiated SH-SY5Y cells (Chi et al. 2019; Sriwastva et al. 2020; Xu et al. 2020a; Zhang et al. 2019b) showed a dose- and time-dependent increase in cell death after hypoxia as measured with flow cytometry or immunofluorescence. Similarly, an increase in cell death after ischemia/hypoxia was measured in three studies with undifferentiated SK-N-SH (Jin et al. 2020; Li et al. 2017; Zhou et al. 2016) and in one study in differentiated SK-N-SH cells (Wang et al. 2020a).

In SH-SY5Y and SK-N-SH cell models, cell death rate after ischemia/hypoxia has been investigated extensively. Eighty-three papers found increased cell death rates after ischemia/hypoxia in undifferentiated SH-SY5Y cells (Castri et al. 2014; Chan et al. 2015; Chang et al. 2017; Chen 2004; Chen et al. 2019; Dong et al. 2019; Fang et al. 2020; Feng et al. 2021; Formisano et al. 2013; Gao et al. 2017, 2020; Guo et al. 2018, 2019; Hong et al. 2017; Hsieh et al. 2021; Hu et al. 2020; Huang et al. 2018, 2021; Janssen et al. 2021; Jia et al. 2021; Jimenez-Almarza et al. 2019; Karuppagounder et al. 2013; Landgraf et al. 2020; Li et al. 2019b, 2020b; Lin-Holderer et al. 2016; Liu 2016; Liu et al. 2016, 2018, 2019b; Lu et al. 2019; Luan et al. 2013; Marmol et al. 2021; Marutani et al. 2012; McCune et al. 2016; Meng et al. 2021; Nampoothiri and Rajanikant 2019; Niu et al. 2018; Park et al. 2017; Roy et al. 2021; Ruan et al. 2020; Shi et al. 2016, 2020, 2021; Sinoy et al. 2017; Song et al. 2019; Tajes et al. 2013; Tan et al. 2021; Tang et al. 2013; Tian et al. 2020; Wang et al. 2013, 2018b, c, 2019a, b, c, 2020b; Wang and Xu 2021; Wen et al. 2020; Wu et al. 2020a, b; Xing et al. 2021; Xu et al. 2020b; Yan et al. 2020; Yang et al. 2014, 2021a, b; Yi et al. 2020; Yin et al. 2021; Zappala et al. 2021; Zhang et al. 2016b, c, 2019a, c, 2020b; Zhao et al. 2013; Zhi et al. 2020; Zhou et al. 2019; Zuo et al. 2020). Eight studies found increased cell death rates after ischemia/hypoxia in differentiated SH-SY5Y cells (Cheng et al. 2019, 2014; Fu et al. 2020; Juntunen et al. 2020; Pan et al. 2020; Sriwastva et al. 2020; Xu et al. 2020a; Zhang et al. 2019b). Four studies found increased cell death rates after ischemia/hypoxia in differentiated and undifferentiated SK-N-SH cell model of cerebral ischemia (Ingrassia et al. 2012; Jin et al. 2020; Yanagita et al. 2005; Yin et al. 2021).

Apoptosis is a possible pathway to cell death. Various pro- and anti-apoptotic factors have been investigated. Apoptosis can be triggered through an intrinsic pathway which is regulated by B-cell lymphoma 2 (BCL-2) protein family and is activated by internal signals. The expression of anti-apoptotic BCL-2 has been investigated in 25 studies using undifferentiated SH-SY5Y cells (Chang et al. 2017; Dong et al. 2019; Feng et al. 2021; Gao et al. 2020; Guo 2019; He et al. 2014; Hu et al. 2020; Huang et al. 2018; Li et al. 2019b, 2020a; Liu et al. 2018, 2020; Wang et al. 2013, 2019c, 2020b, 2021a, b; Wu et al. 2020a; Xu et al. 2020b; Yan et al. 2020; Zhang et al. 2016b; Zhao and Wang 2020; Zhi et al. 2020; Zhou et al. 2019) and in one study using differentiated SH-SY5Y cells (Sriwastva et al. 2020). It was found that BCL-2 decreased during and after ischemia/hypoxia. One study investigated the effect of hypoxia on phosphorylated BCL-2 in undifferentiated SH-SY5Y cells and showed increased levels of phosphorylated BCL-2, which could induce autophagic cell death (Wang et al. 2019c). A second member of the BCL-2 family, B-cell lymphoma-extra-large (BCL-XL), an anti-apoptotic protein like BCL-2, prevents apoptosis by preventing the release of small mitochondrial molecules like cytochrome-c (cyt-c). BCL-XL expression was inhibited by ischemia/hypoxia in undifferentiated SH-SY5Y cell models (n = 4) (Castri et al. 2014; He et al. 2014; Li et al. 2020a; Wang et al. 2021b). Besides BCL-2 and BCL-XL, a third anti-apoptotic factor of the BCL-2 family has been investigated, namely Bcl-2-like protein 2 (BCL-W). BCL-W decreased during hypoxia in differentiated SH-SY5Y cells (n = 1) (Xu et al. 2020a).

In addition to anti-apoptotic factors, the BCL-2 family has members that act as pro-apoptotic factors, such as BCL2 associated X (BAX). Expression of this pro-apoptotic protein increased in undifferentiated SH-SY5Y cell models of cerebral ischemia (n = 23) (Castri et al. 2014; Dong et al. 2019; Feng et al. 2021; Gao et al. 2020; Guo 2019; He et al. 2014; Hu et al. 2020; Huang et al. 2018; Li et al. 2019b, 2020a; Liu et al. 2018, 2020; Wang et al. 2013, 2018a, 2019c, 2021b; Wu et al. 2020a; Xu et al. 2020b; Yan et al. 2020; Zhang et al. 2016b; Zhao and Wang 2020; Zhou et al. 2019). Expression of BAX is only investigated in one study using differentiated SH-SY5Y cells. These results showed increased expression of BAX during and after hypoxia (Sriwastva et al. 2020). BAX induces cell death via mitochondrial membrane permeabilization. This results in the release of small molecules such as cyt-c and apoptosis inducing factor (AIF), among others.

Release of cyt-c is investigated in four studies in undifferentiated SH-SY5Y cells (Hu et al. 2020; Li et al. 2020a; Lin et al. 2020; Wang et al. 2019b). The results showed increased cyt-c release, which led to increased apoptotic rate. Bcl2-interacting killer (BIK) enhances apoptosis, it was found to be upregulated in undifferentiated SH-SY5Y cells (Chen et al. 2019). Another pro-apoptotic protein of the BCL-2 family has been investigated in undifferentiated SH-SY5Y cells, namely Bcl-2-like protein 11 (BIM). The expression of BIM was found to be increased during ischemia/hypoxia (n = 2) (He et al. 2014; Song et al. 2019). In addition to BIM, apoptosis facilitator Bcl-2-like protein 14 (BCL2L14) has been investigated in undifferentiated SH-SY5Y cells and the results showed increased expression, which induced apoptosis during ischemia/hypoxia (Yao et al. 2019).

The BCL-2 family induces or prevents the release of apoptogenic factors, which can lead to the activation of the caspase pathway. The caspase family is extensively investigated in relation to apoptosis in ischemia/hypoxia. In studies with undifferentiated SH-SY5Y cells (n = 39), two members of the caspase family (caspase-3 and caspase-9) were increased during and after ischemia/hypoxia (Castri et al. 2014; Chang et al. 2017; Chen et al. 2015, 2019; Dong et al. 2019; Du et al. 2012; Feng et al. 2021; Gao et al. 2020; Hong et al. 2017; Huang et al. 2018; Jia et al. 2021; Li and Ma 2020; Lin et al. 2020; Liu et al. 2018; Marutani et al. 2012; Park et al. 2017; Ruan et al. 2020; Shan et al. 2022; Shi et al. 2020; Song et al. 2019; Tan et al. 2021; Wang et al. 2018a, c, 2019b, 2021a, b; Wen et al. 2020; Wu et al. 2020a; Xing et al. 2021; Xu et al. 2020b; Yan et al. 2020; Yi et al. 2020; Yin et al. 2021; Zappala et al. 2021; Zhang et al. 2016a, b; Zhao and Wang 2020; Zhi et al. 2020; Zuo et al. 2020). Increased expression of caspase-3, -6, -7 after ischemia/hypoxia was found in differentiated SH-SY5Y (n = 5) (Cheng et al. 2014; Sriwastva et al. 2020; Xu et al. 2020a; Zhang et al. 2019b; Zhou et al. 2014). Pro-caspase-9 was found to be negatively correlated with X-linked inhibitor of apoptosis in undifferentiated SH-SY5Y cells (n = 1) (Zhang et al. 2016a). In three studies using undifferentiated SK-N-SH cells, increased expression of caspase-1, -3 and -7 was found after ischemia/hypoxia (Lehane et al. 2013; Yin et al. 2021; Zhou et al. 2016). Similarly, increased expression of caspase-3 was found in one study with differentiated SK-N-SH (Wang et al. 2020a).

AIF translocate to the nucleus upon release by mitochondria, where it triggers chromatin condensation and DNA fragmentation to induce parthanatos. AIF translocation is investigated in undifferentiated SH-SY5Y cells (n = 2). The results showed nuclear translocation of AIF induced by ischemia/hypoxia (Li et al. 2020a; Wang et al. 2018a). One study investigated the expression of AIF in differentiated SH-SY5Y cells and the results showed increased expression induced by hypoxia (Sriwastva et al. 2020). In addition to AIF release from mitochondria, poly(ADP-ribose) polymerase-1 (PARP-1) overactivation mediates AIF release. Normal function of PARP-1 is DNA repair. Cleaved PARP induces parthanatos, which has been investigated in undifferentiated SH-SY5Y cells (n = 5), differentiated SH-SY5Y cells (n = 3), and undifferentiated SK-N-SH (n = 1). Cleaved PARP was increased, which was in turn associated with ischemia/hypoxia induced parthanatos (Han et al. 2017; Lehane et al. 2013; Li et al. 2020a; Ruan et al. 2020; Shi et al. 2020; Sriwastva et al. 2020; Wang et al. 2018a; Xing et al. 2021; Xu et al. 2020a). Endothelial protein C receptor (EPCR) showed anti-cell death features by signaling via protease-activated receptor 1 (PAR1) and PAR3. Levels of these receptors were decreased in differentiated SH-SY5Y cells during and after ischemia/hypoxia (n = 1) (Sriwastva et al. 2020).

Another key player in apoptosis is the tumor suppressor gene *P53*. This gene is activated by hypoxia and can induce apoptosis. *P53* is investigated in one study using undifferentiated SH-SY5Y cells and showed that *P53* activity was induced by ischemia/hypoxia (Zhao et al. 2013).

PI3K/AKT/mTOR pathway is an intracellular signaling pathway that plays a role in regulating the cell cycle and apoptosis. This has been investigated in sixteen studies using undifferentiated SH-SY5Y cell models of cerebral ischemia. Overall, a decrease in phosphorylated protein kinase B (AKT), phosphorylated phosphoinositide 3-kinases (PI3K), mTOR and forkhead box proteins (FOXO) were found (Feng et al. 2020; Gao et al. 2020; Guo et al. 2018; Li et al. 2019b; Liu et al. 2019b, 2021; Marutani et al. 2012; Song et al. 2019; Tian et al. 2020; Wang et al. 2019b; Wen et al. 2020; Wu et al. 2020a; Yi et al. 2020; Zhi et al. 2020; Zhou et al. 2019). Decreased levels of AKT were also found in two studies using differentiated SH-SY5Y cell models (Cheng et al. 2019; Han et al. 2017). Epidermal growth factor receptor showed anti-apoptotic effects by activation of the PI3K/AKT pathway in one study with undifferentiated SH-SY5Y and one study with differentiated SH-SY5Y cell models of cerebral ischemia (Lin et al. 2011; Wu et al. 2020b). Glycogen synthase kinase 3β (GSK-3β) was found to be dependent on several signaling pathways, like PI3K/AKT. One study in differentiated SH-SY5Y cells found that increased levels of GSK-3β led to increased apoptotic rate (Lin et al. 2011). Upregulation of phosphatase and tensin homolog (PTEN) was found in four undifferentiated SH-SY5Y and one undifferentiated SK-N-SH cell models of cerebral ischemia (Gao et al. 2020; Guo 2019; Guo et al. 2018; Jin et al. 2020; Yi et al. 2020).

Some other factors that are associated with cell death and have been investigated in human neuronal in vitro models of cerebral ischemia are Sphingosine kinase 2 (decreased with ischemia in undifferentiated SH-SY5Y cells) (n = 1) (Di et al. 2017), death-associated protein kinase 1 (DAPK1; elevated with ischemia in undifferentiated SH-SY5Y cells) (n = 1; (Feng et al. 2021)), heat shock protein20 (HSP20; decreased and initiated Golgi apparatus fragmentation) (n = 1) (Lu et al. 2019), and matrix metallopeptidase 9 (MMP-9; increased macrophage migration inhibitory factor (MIF), which is recruited to cleave DNA) (n = 1) (Li et al. 2020a).

Increased nuclear fragmentation has been found in undifferentiated SH-SY5Y cell model (n = 1) (Liu et al. 2019a). FMS-like tyrosine kinase-3 (FLT3) has been implicated in cell survival. One study into this kinase showed that FLT3 expression increased in undifferentiated SH-SY5Y cell model of cerebral ischemia (Dong et al. 2019).

Toll-like receptor 8 (TLR8) was correlated with increased cell death rate in an undifferentiated SH-SY5Y cell model (Tang et al. 2013). Levels of brain derived neurotrophic factor (BDNF) showed no changes during ischemia/hypoxia in undifferentiated SH-SY5Y cells (n = 3) (Chang et al. 2017; Fang et al. 2020; Wang et al. 2013). The MAPK/JNK/ERK signaling pathway has been implicated in regulation of cell death in differentiated (n = 4) (Cheng et al. 2014; Chi et al. 2019; Xu et al. 2020a; Zeng et al. 2019) and undifferentiated SH-SY5Y (n = 13) cell models of cerebral ischemia (He et al. 2014; Liu et al. 2019a; Marutani et al. 2012; Ruan et al. 2021; Shi et al. 2020; Tian et al. 2020; Wang et al. 2013, 2017, 2019c; Yang et al. 2014). Interferon regulatory factor-1 (IRF-1) has been investigated in relation to cell death and results showed a correlation between increased IRF-1 and cell death in undifferentiated SH-SY5Y cell model of cerebral ischemia (n = 1) (Liu et al. 2020).

The pro-apoptotic factor ‘programmed cell death 4’ (PDCD4) was found to be increased in an undifferentiated SH-SY5Y cell model of cerebral ischemia (Shan et al. 2022). Increased expression of inhibitor of growth protein 5 (ING5) was correlated with an increased apoptotic rate in an undifferentiated SH-SY5Y cell model of cerebral ischemia (Zhang et al. 2019a). Hypoxia-inducible factor-1 showed to be upregulated, followed by downregulation during ischemia/hypoxia and played a role in ischemia/hypoxia induced cell death in undifferentiated SH-SY5Y cells (n = 4) and SK-N-SH cells (n = 1) (Lin-Holderer et al. 2016; Niu et al. 2018; Olechnowicz et al. 2012; Ruan et al. 2021; Zhang et al. 2016c). Modulation of apoptosis-1 is an apoptotic pathway that was found to be increased in undifferentiated SH-SY5Y cells (n = 1) (Chan et al. 2019).

Cell membrane damage has been investigated in differentiated and undifferentiated SH-SY5Y cell models (n = 28) (Chan et al. 2015; Chen et al. 2019; Cheng et al. 2014, 2019; Chi et al. 2019; Gao et al. 2020; Hsieh et al. 2021; Li and Ma 2020; Lin et al. 2011; Luan et al. 2013; Marutani et al. 2012; Nampoothiri and Rajanikant 2019; Park et al. 2017; Ruan et al. 2020; Sriwastva et al. 2020; Wang et al. 2018a, 2019b, c; Wang and Xu 2021; Wu et al. 2020a, b; Xing et al. 2021; Xu et al. 2020a; Zeng et al. 2019; Zhang et al. 2016c, 2019b; Zhao et al. 2013; Zhi et al. 2020) and differentiated and undifferentiated SK-N-SH cell models (n = 3) (Ingrassia et al. 2012; Lehane et al. 2013; Rosenthal et al. 2017).

#### NT2-N Cell Model

Two studies investigated ischemia/hypoxia in differentiated NTERA-2 clone D1 (NT2-N) cell model. These studies investigated complement system activation upon ischemia/hypoxia induced stress. The complement system is part of the immune system that promotes inflammation. The first study into expression of several complement regulators, such as CD35, CD45, CD55, CD59, C3a and C5a, showed decreased expression of CD55 and an increased vulnerability to hypoxia (Pedersen et al. 2007b).

The second study aimed to investigate whether complement regulatory protein CD59 was expressed in the NT2-N cell model and what its function was. The results showed that CD59 was expressed and that this protein played a role in protecting the cells from complement attack and eventually cell death (Pedersen et al. 2007a).

#### hiPSC-Derived Neuronal Cell Model

Two studies investigated ischemia/hypoxia in healthy hiPSC-derived neuronal cell models. One study investigated hiPSC-derived neuronal networks consisting of excitatory and inhibitory neurons. Electrophysiological measurements showed decreased neuronal activity, as well as decreased synchronous activity, throughout the network. Furthermore, the results showed increased levels of apoptosis on time scales of 24–48 h of hypoxia, followed by an increased level of dead cells (Pires Monteiro et al. 2021).

The other study compared the effect of ischemia/hypoxia on hiPSC-derived neurons with that on differentiated SH-SY5Y cells. The results showed less cell survival of SH-SY5Y cells than of hiPSC-derived neurons (Juntunen et al. 2020).

### Treatment Targets

Several steps of the ischemic cascade have been targeted by pharmacological treatment strategies to prevent irreversible neuronal damage. Thirty-six studies in undifferentiated SH-SY5Y cell models (Chen et al. 2021b; Dong et al. 2021; Gao et al. 2017; Hao et al. 2015; Hong et al. 2017; Hsieh et al. 2021; Huang et al. 2021; Janssen et al. 2021; Jiang et al. 2012; Jimenez-Almarza et al. 2019; Lai et al. 2020; Landgraf et al. 2020; Li et al. 2019a, b, 2020a; Lin-Holderer et al. 2016; Liu et al. 2016, 2019a, b; Liu 2016; Luan et al. 2013; Marutani et al. 2012; Park et al. 2017; Ruan et al. 2021; Shi et al. 2019; Song et al. 2019; Wang et al. 2013, 2019b, c, 2021a; Wu et al. 2020a, b; Xu et al. 2020b; Yang et al. 2014; Zappala et al. 2021; Zhi et al. 2020), seven in differentiated SH-SY5Y cell models (Cheng et al. 2014, 2019; Fu et al. 2020; Lin et al. 2011; Sriwastva et al. 2020; Zeng et al. 2019; Zhang et al. 2019b), two in undifferentiated SK-N-SH cell models (Lehane et al. 2013; Soh et al. 2007), and one in hiPSC-derived neurons (Pires Monteiro et al. 2021) included treatment with a pharmacological compound. Treatment targets were neuronal activity, oxidative/nitrosative stress, inflammation, autophagy or cell death.

One study with hiPSC-derived neurons investigated the effect of neuronal network activation by the mildly excitatory hormone/neurotransmitter ghrelin (Pires Monteiro et al. 2021). The results showed partial preservation of neuronal network functioning after hypoxia (Pires Monteiro et al. 2021).

Oxidative/nitrosative stress was investigated as a treatment target in twenty studies using undifferentiated SH-SY5Y cells and one study using differentiated SH-SY5Y cells. These studies showed that various chemical compounds could reduce oxidative stress by reducing the formation of ROS or increasing anti-oxidant defense mechanisms, which led to increased cell survival (Agudo-Lopez et al. 2010; Chen et al. 2021b; Dong et al. 2021; Gao et al. 2017; Hong et al. 2017; Hsieh et al. 2021; Jimenez-Almarza et al. 2019; Landgraf et al. 2020; Li et al. 2020a; Liu et al. 2019a; Liu 2016; Marutani et al. 2012; Park et al. 2017; Ruan et al. 2021; Shi et al. 2019; Sriwastva et al. 2020; Wang et al. 2019b; Wu et al. 2020a; Xu et al. 2020b; Yang et al. 2021b; Zhi et al. 2020).

Inflammation was investigated as a treatment target in twelve studies using undifferentiated SH-SY5Y cells and in one study using differentiated SH-SY5Y cells. These studies showed that various chemical compounds could decrease several pro-inflammatory factors (NF-κB, TNF, IL-6, IL-B1) to alleviate inflammation (Chen et al. 2015; Dong et al. 2021; Hao et al. 2015; Huang et al. 2021; Janssen et al. 2021; Jiang et al. 2012; Landgraf et al. 2020; Li et al. 2019a, b; Ruan et al. 2021; Yang et al. 2014; Zhi et al. 2020).

Autophagy was investigated as a treatment target in five studies using undifferentiated SH-SY5Y cells and two studies using differentiated SH-SY5Y cells (Cheng et al. 2019; Lai et al. 2020; Liu 2016; Liu et al. 2016; Wang et al. 2019c; Zhang et al. 2019b; Zhi et al. 2020). These studies showed that various pharmacological compounds could maintain healthy autophagic flux.

Apoptosis was investigated as a treatment target in 26 studies using undifferentiated SH-SY5Y cells, four studies using differentiated SH-SY5Y cells, and two studies using undifferentiated SK-N-SH cells. These studies showed that pharmacological compounds could decrease apoptosis, by reducing expression of pro-apoptotic factors and increasing expression of anti-apoptotic factors, which led to increased cell survival (Agudo-Lopez et al. 2010; Chen et al. 2021b; Cheng et al. 2014; Dong et al. 2021; Gao et al. 2017; Hao et al. 2015; Huang et al. 2021; Jiang et al. 2012; Li et al. 2019b, 2020a; Lin et al. 2011, 2020; Liu et al. 2019a, b; Luan et al. 2013; Marutani et al. 2012; Park et al. 2017; Song et al. 2019; Sriwastva et al. 2020; Wang et al. 2013, 2019b, c, 2021a; Wu et al. 2020a, b; Xu et al. 2020b; Yang et al. 2014; Zappala et al. 2021; Zhang et al. 2019b; Zhi et al. 2020).

## Discussion

With this scoping review, we provide an overview of human neuronal in vitro models that have been used to study human neuronal responses to ischemia or hypoxia, including the established steps in the pathophysiological cascade and potential treatment targets. The scoping review shows that models based on SH-SY5Y cells are the most widely used in this field. Almost all studies used immunocytochemical techniques to investigate cell survival and protein expression. Neuronal functioning was investigated in only one study using hiPSC-derived neuronal networks. Cell death was the most investigated pathomechanism, followed by oxidative stress and inflammation. Studies on apoptosis revealed the importance of diverse pro- and anti-apoptotic factors such as the BCL-2 protein family that lead to apoptosis and eventually cell death. Studies on oxidative stress showed higher levels of ROS and lower levels of ROS scavenging molecules after ischemia or hypoxia. The most important results on inflammatory responses were increased levels of pro-inflammatory and decreased levels of anti-inflammatory cytokines.

SH-SY5Y cells are a subclone from SK-N-SH, originating from a single 4-year-old female neuroblastoma patient (Biedler et al. 1973). The wide spread use of SH-SY5Y cells indicates that pathomechanisms of human neuronal responses to ischemia or hypoxia have mainly been investigated in a cancerous cell line (Liu et al. 2018). It is known that the genetic background of a cell line affects functionality, as well as responses to interventions (Mossink et al. 2021b). Thus, a cell line with cancerous characteristics will probably respond to ischemia or hypoxia differently than healthy (neuronal) cells.

Moreover, although SH-SY5Y cells can be differentiated into neuron-like cells upon stimulation with RA (Khwanraj et al. 2015; Korecka et al. 2013; Shipley et al. 2016), the majority of studies has been conducted in undifferentiated SH-SY5Y cells. Undifferentiated and differentiated SH-SY5Y cells differ in morphology and function. Undifferentiated SH-SY5Y cells have large cell bodies with short truncated processes (Kovalevich and Langford 2013). These undifferentiated cells express immature neuronal markers, such as SOX2, and lack the expression of mature neuronal markers, such as synapsin1/2 positive puncta. Differentiated SH-SY5Y cells form and extent neuritic processes (Kovalevich and Langford 2013) and show increased expression of synaptic markers, such as SNAP25 and SYN1 (Forster et al. 2016). Differentiated SH-SY5Y cells acquired the neuronal ability to produce trains of spikes upon prolonged stimulation in current-clamp experiments (Tosetti et al. 1998) and showed spontaneous firing, bursting, and network behaviour on micro-electrode arrays (MEAs) (Yoon et al. 2020), that were not observed in undifferentiated SH-SY5Y cells. These differences may lead to divergent responses to ischemia/hypoxia between undifferentiated and differentiated SH-SY5Y cells. Thus, it is questionable whether results from studies in undifferentiated SH-SY5Y cells can be extrapolated to neurons or neuronal networks.

OGD directly models ischemic stress and allows for the exploration of divergent components of the pathophysiological cascade caused by ischemic stress. Furthermore, modelling of OGD is straightforward and can be replicated in various laboratories (Cimarosti and Henley 2008). Other proposed cerebral ischemia models are based on chemical or enzymatic modulation of specific pathophysiological pathways. These can be used to study specific components of the ischemic cascade, such as excitotoxicity or glutathione depletion. These techniques are commonly used to investigate the effect of increased glutamate release or induced oxidative stress caused by ischemic stroke, respectively (Nicholls and Ward 2000). In turn, these may lead to other downstream effects, for example calcium dysregulation (by excitotoxicity) or glutathione depletion (by oxidative stress) (Mattson et al. 1995; Mytilineou et al. 1998). However, it is important to note that these techniques probably model only parts of the ischemic stress cascade (Cimarosti and Henley 2008). Moreover, it is unclear whether chemical or enzymatic induction of downstream effects has the desired effect on undifferentiated SH-SY5Y cells, which are often used in ischemic stroke research. For example, none of the studies included in this scoping review touched upon excitotoxicity. This highlights the need for a careful assessment of the suitability of the various cell models and modes of ischemic stress induction in light of the research question at hand.

The majority of human neuronal in vitro studies on effects of ischemia/hypoxia focused on cell survival and protein expression. Expression of proteins that might have deleterious effects (such as pro-apoptotic factors, pro-inflammatory cytokines, oxidative stress factors) apparently increase and expression of proteins that might have protective effects (such as anti-apoptotic factors and anti-inflammatory factors) decrease. Various studies showed this in relationship to cell death, apoptosis, oxidative stress, or inflammation. This focus on microscopic measures of relatively downstream processes neglects early steps in the pathophysiological cascade of ischemia, such as energy failure caused by OGD (Galkin 2019), loss of cell ion homeostasis (Caplan 2009; Taoufik and Probert 2008), and depression of excitatory synaptic transmission. It also neglects effects on neuronal functionality and activity (Bolay et al. 2002; Hofmeijer and van Putten 2012; Pires Monteiro et al. 2021).

HiPSC-derived neuronal networks on MEAs are currently the only functional human neuronal cell model used to investigate neuronal network activity in cerebral ischemia (Pires Monteiro et al. 2021). HiPSCs can be derived from healthy individuals or patients and differentiated in excitatory neurons and inhibitory neurons. This allows establishment of healthy neuronal networks with physiological excitatory – inhibitory (E/I) ratio’s (Frega et al. 2017; Mossink et al. 2021a). Genetic characteristics of the donor are preserved in the model, making it possible to study patient-specific network behaviour, including responses to ischemia/hypoxia (Mossink et al. 2021b). By the use of MEAs, functionality of many neurons and synapses in the network can be studied simultaneously. Despite the lack of normal brain architecture, neuronal network functioning can be readily derived, in addition to microscopic measures. The dynamics of neuronal network failure, from reversible to irreversible damage or recovery, can be monitored continuously (Pires Monteiro et al. 2021). This allows identification of early determinants of irreversibility, including possible effects of interventions.

Studies on interventions to ameliorate pathophysiological processes during or after ischemia/hypoxia focused on a wide range of treatment targets. Evidence from studies in SH-SY5Y cells mostly suggests effects of treatments targeting apoptosis or other modes of cell death, because this was the most targeted pathomechanism (Agudo-Lopez et al. 2010; Chen et al. 2021b; Cheng et al. 2014; Dong et al. 2021; Gao et al. 2017; Hao et al. 2015; Huang et al. 2021; Jiang et al. 2012; Li et al. 2019b, 2020a; Lin et al. 2011, 2020; Liu et al. 2019a, b; Luan et al. 2013; Marutani et al. 2012; Park et al. 2017; Song et al. 2019; Sriwastva et al. 2020; Wang et al. 2013, 2019b, c, 2021a; Wu et al. 2020a, b; Xu et al. 2020b; Yang et al. 2014; Zappala et al. 2021; Zhang et al. 2019b; Zhi et al. 2020). One study in hiPSC-derived neuronal networks on MEAs showed beneficial effects of early neuronal network stimulation on functional network recovery and cell survival (Pires Monteiro et al. 2021). All intervention studies were small sampled proof of principle experiment and none of the intervention effects was conformed in follow-up studies.

In vitro models of OGD provide a versatile platform for replicating diverse aspects of ischemic stroke pathology in a reproducible manner. Compared to in vivo models, these models are often quicker, cheaper and easier to establish. Also, the use of human-derived models may contribute to a reduction of animal model use. However, for clinical translation, combinations of in vitro and in vivo models will continue to be necessary (Trotman-Lucas and Gibson 2021). With the increasing amount of modelling possibilities, it will remain important to consider the optimal model based on the research goal at hand.

The strengths of this scoping review are the prospective literature research and systematic data extraction. Limitations include that the review was not pre-registered, data extraction was conducted by only one reviewer (EV), and lack of quality assessment of the included articles. Data on effort, costs, and reproducibility of the models are not systematically presented in the included research papers.

In conclusion, the majority of human neuronal in vitro studies on experimental cerebral ischemia/hypoxia exploited the cancerous SH-SY5Y cell line, mostly in a relatively immature stage and not differentiated into neuronal-like cells. Most studies focused on microscopic measures of single downstream pathophysiological processes, such as cell death. Intervention studies targeted a myriad of treatment targets and none of the intervention effects presented in this review have been confirmed in follow-up experiments. We suggest the use of networks of differentiated neurons on MEAs. These provide new opportunities to analyze neuronal network functioning, in addition to microscopic measures. This allows coupling of neuronal structure and function (which is not possible in unfunctional neurons) in order to identify drivers of neuronal network (dys)functioning. The use of hiPSC-derived neuronal networks provides additional opportunities to study networks with physiological E/I ratios and identify person-specific characteristics. For intervention studies, we favor standardization to collect strong evidence of (in)efficacy to improve translation from bench to bedside.

## Supplementary Information

Below is the link to the electronic supplementary material.

Supplementary file1 (DOCX 53 KB)

## Data Availability

Enquiries about data availability should be directed to the authors.
