# Supplementary material for: Neuronal Responses to Ischemia: Scoping Review of Insights from Human-Derived In Vitro Models
Source: Cell Mol Neurobiol. 2023 Jun 28;43(7):3137–60. doi: 10.1007/s10571-023-01368-y (PMC10477161; doi:10.1007/s10571-023-01368-y)
Supplement: Supplementary file 1 — Supplementary file1 (DOCX 53 KB) [file 10571_2023_1368_MOESM1_ESM.docx]

**Supplementary materials**

1. Supplementary figure 1. Flow chart
2. Supplementary reference list.
3. **Supplementary figure 1. Flow chart**

**Identification of studies via databases and registers**

Records removed *before screening*: 0

Records identified by literature search

(n = 278)

**Identification**

Records excluded: 125

Reasons for exclusion: wrong cell model, wrong mode of ischemia modelling, treatment before induction of OGD

Records screened based on title and abstract

(n = 278)

Reports sought for retrieval

(n = 153)

Reports not retrieved: 6

**Screening**

Reports excluded: 0

Reports assessed for eligibility

(n = 147)

Studies included in review

(n = 147)

**Included**

1. **Supplementary reference list corresponding to table 1 and table 2.**

1. Beske, P.H. and D.A. Jackson, *NADPH oxidase mediates the oxygen-glucose deprivation/reperfusion-induced increase in the tyrosine phosphorylation of the N-methyl-D-aspartate receptor NR2A subunit in retinoic acid differentiated SH-SY5Y Cells.* J Mol Signal, 2012. **7**(1): p. 15.

2. Cheng, A., et al., *Attenuating oxygen-glucose deprivation-caused autophagosome accumulation may be involved in sevoflurane postconditioning-induced protection in human neuron-like cells.* Eur J Pharmacol, 2019. **849**: p. 84-95.

3. Cheng, Y.L., et al., *Evidence that neuronal Notch-1 promotes JNK/c-Jun activation and cell death following ischemic stress.* Brain Res, 2014. **1586**: p. 193-202.

4. Chi, L., et al., *miR-9-5p attenuates ischemic stroke through targeting ERMP1-mediated endoplasmic reticulum stress.* Acta Histochem, 2019. **121**(8): p. 151438.

5. Fu, C., et al., *Neuroprotective Effects of Qingnao Dripping Pills Against Cerebral Ischemia via Inhibiting NLRP3 Inflammasome Signaling Pathway: In Vivo and In Vitro.* Front Pharmacol, 2020. **11**: p. 65.

6. Han, J., B. Luk, and F.J. Lee, *Neuroprotective effects of extracellular DJ-1 on reperfusion injury in SH-SY5Y cells.* Synapse, 2017. **71**(5).

7. Juntunen, M., et al., *In Vitro Oxygen-Glucose Deprivation-Induced Stroke Models with Human Neuroblastoma Cell- and Induced Pluripotent Stem Cell-Derived Neurons.* Stem Cells Int, 2020. **2020**: p. 8841026.

8. Lin, D., G. Li, and Z. Zuo, *Volatile anesthetic post-treatment induces protection via inhibition of glycogen synthase kinase 3beta in human neuron-like cells.* Neuroscience, 2011. **179**: p. 73-9.

9. Pan, H., et al., *Overexpression of long non-coding RNA SNHG16 against cerebral ischemia-reperfusion injury through miR-106b-5p/LIMK1 axis.* Life Sci, 2020. **254**: p. 117778.

10. Sriwastva, M.K., et al., *Neuroprotective Effects of Activated Protein C Involve the PARP/AIF Pathway against Oxygen-Glucose Deprivation in SH-SY5Y Cells.* Brain Sci, 2020. **10**(12).

11. Xu, S., et al., *Oxygen glucose deprivation/re-oxygenation-induced neuronal cell death is associated with Lnc-D63785 m6A methylation and miR-422a accumulation.* Cell Death Dis, 2020. **11**(9): p. 816.

12. Zeng, Z., et al., *Qingnao dripping pills mediate immune-inflammatory response and MAPK signaling pathway after acute ischemic stroke in rats.* J Pharmacol Sci, 2019. **139**(3): p. 143-150.

13. Zhang, X., et al., *Autophagy Induced by Oxygen-Glucose Deprivation Mediates the Injury to the Neurovascular Unit.* Med Sci Monit, 2019. **25**: p. 1373-1382.

14. Zhou, M., et al., *Cold-inducible RNA-binding protein mediates neuroinflammation in cerebral ischemia.* Biochim Biophys Acta, 2014. **1840**(7): p. 2253-61.

15. Agudo-Lopez, A., et al., *Involvement of mitochondria on neuroprotective effect of sphingosine-1-phosphate in cell death in an in vitro model of brain ischemia.* Neurosci Lett, 2010. **470**(2): p. 130-3.

16. Castri, P., et al., *Poly(ADP-ribose) polymerase-1 and its cleavage products differentially modulate cellular protection through NF-kappaB-dependent signaling.* Biochim Biophys Acta, 2014. **1843**(3): p. 640-51.

17. Chai, Z., et al., *Inhibition of miR-19a-3p decreases cerebral ischemia/reperfusion injury by targeting IGFBP3 in vivo and in vitro.* Biol Res, 2020. **53**(1): p. 17.

18. Chan, S.J., et al., *Cystathionine beta-synthase inhibition is a potential therapeutic approach to treatment of ischemic injury.* ASN Neuro, 2015. **7**(2).

19. Chan, S.J., et al., *Modulator of apoptosis-1 is a potential therapeutic target in acute ischemic injury.* J Cereb Blood Flow Metab, 2019. **39**(12): p. 2406-2418.

20. Chang, C.F., et al., *(-)-Phenserine inhibits neuronal apoptosis following ischemia/reperfusion injury.* Brain Res, 2017. **1677**: p. 118-128.

21. Chen, B., et al., *Calreticulin Binds to Fas Ligand and Inhibits Neuronal Cell Apoptosis Induced by Ischemia-Reperfusion Injury.* Biomed Res Int, 2015. **2015**: p. 895284.

22. Chen, S.H.C., R. T. F., *Neuropeptide Y-Y1 Receptor Agonist Worsens while Antagonist Improves Survival of Cultured Y1-Expressing Neuronal Cells following Oxygen and Glucose Deprivation.* Journal of Biomedical Science, 2004. **11**: p. 781-788.

23. Chen, W., L. Wang, and Z. Liu, *MicroRNA-155 influences cell damage in ischemic stroke via TLR4/MYD88 signaling pathway.* Bioengineered, 2021. **12**(1): p. 2449-2458.

24. Chen, X., et al., *Upregulation of miR-1306-5p decreases cerebral ischemia/reperfusion injury in vitro by targeting BIK.* Biosci Biotechnol Biochem, 2019. **83**(12): p. 2230-2237.

25. Chen, Y., Z. Fan, and Q. Wu, *Dexmedetomidine improves oxygen-glucose deprivation/reoxygenation (OGD/R) -induced neurological injury through regulating SNHG11/miR-324-3p/VEGFA axis.* Bioengineered, 2021. **12**(1): p. 4794-4804.

26. Di, G., et al., *AntagomiR-613 protects neuronal cells from oxygen glucose deprivation/re-oxygenation via increasing SphK2 expression.* Biochem Biophys Res Commun, 2017. **493**(1): p. 188-194.

27. Di, G., et al., *CEBPA-AS1 Knockdown Alleviates Oxygen-Glucose Deprivation/Reperfusion-Induced Neuron Cell Damage by the MicroRNA 24-3p/BOK Axis.* Mol Cell Biol, 2021. **41**(8): p. e0006521.

28. Dong, R.F., et al., *Neuroprotective effect of FMS-like tyrosine kinase-3 silence on cerebral ischemia/reperfusion injury in a SH-SY5Y cell line.* Gene, 2019. **697**: p. 152-158.

29. Dong, X., et al., *Physcion Protects Rats Against Cerebral Ischemia-Reperfusion Injury via Inhibition of TLR4/NF-kB Signaling Pathway.* Drug Des Devel Ther, 2021. **15**: p. 277-287.

30. Du, C.P., R. Tan, and X.Y. Hou, *Fyn kinases play a critical role in neuronal apoptosis induced by oxygen and glucose deprivation or amyloid-beta peptide treatment.* CNS Neurosci Ther, 2012. **18**(9): p. 754-61.

31. Fang, Y.C., et al., *HDAC inhibitor protects chronic cerebral hypoperfusion and oxygen-glucose deprivation injuries via H3K14 and H4K5 acetylation-mediated BDNF expression.* J Cell Mol Med, 2020. **24**(12): p. 6966-6977.

32. Feng, H., et al., *Repurposing antimycotic ciclopirox olamine as a promising anti-ischemic stroke agent.* Acta Pharm Sin B, 2020. **10**(3): p. 434-446.

33. Feng, M., X. Zhu, and C. Zhuo, *H19/miR-130a-3p/DAPK1 axis regulates the pathophysiology of neonatal hypoxic-ischemia encephalopathy.* Neurosci Res, 2021. **163**: p. 52-62.

34. Formisano, L., et al., *NCX1 is a new rest target gene: role in cerebral ischemia.* Neurobiol Dis, 2013. **50**: p. 76-85.

35. Gao, G.S., et al., *Humanin analogue, S14G-humanin, has neuroprotective effects against oxygen glucose deprivation/reoxygenation by reactivating Jak2/Stat3 signaling through the PI3K/AKT pathway.* Exp Ther Med, 2017. **14**(4): p. 3926-3934.

36. Gao, N., et al., *LncRNA H19 Aggravates Cerebral Ischemia/Reperfusion Injury by Functioning as a ceRNA for miR-19a-3p to Target PTEN.* Neuroscience, 2020. **437**: p. 117-129.

37. Guo, S., et al., *Specific inhibition of hypoxia inducible factor 1 exaggerates cell injury induced by in vitro ischemia through deteriorating cellular redox environment.* J Neurochem, 2009. **108**(5): p. 1309-21.

38. Guo, X.-L.L., H.; Sun, Y.; Wang, C.-B, *MicroRNA-26a regulates cerebral ischemia injury through targeting PTEN.* European Review for Medical and Pharmacological Sciences, 2019. **23**: p. 7033-7041.

39. Guo, Y., et al., *Effects of Ginsenoside Rb1 on Expressions of Phosphorylation Akt/Phosphorylation mTOR/Phosphorylation PTEN in Artificial Abnormal Hippocampal Microenvironment in Rats.* Neurochem Res, 2018. **43**(10): p. 1927-1937.

40. Hao, M., et al., *Triptolide Protects Against Ischemic Stroke in Rats.* Inflammation, 2015. **38**(4): p. 1617-23.

41. He, C., A.R. Stroink, and C.X. Wang, *The role of DAPK-BimEL pathway in neuronal death induced by oxygen-glucose deprivation.* Neuroscience, 2014. **258**: p. 254-62.

42. Herrmann, A.G., et al., *Adaptive changes in the neuronal proteome: mitochondrial energy production, endoplasmic reticulum stress, and ribosomal dysfunction in the cellular response to metabolic stress.* J Cereb Blood Flow Metab, 2013. **33**(5): p. 673-83.

43. Hong, S., et al., *Mulberrofuran G Protects Ischemic Injury-induced Cell Death via Inhibition of NOX4-mediated ROS Generation and ER Stress.* Phytother Res, 2017. **31**(2): p. 321-329.

44. Hsieh, Y.S., Y.K. Shin, and G.H. Seol, *Protection of the neurovascular unit from calcium-related ischemic injury by linalyl acetate.* Chin J Physiol, 2021. **64**(2): p. 88-96.

45. Hu, L., R. Fang, and M. Guo, *Knockdown of lncRNA SNHG1 alleviates oxygen-glucose deprivation/reperfusion-induced cell death by serving as a ceRNA for miR-424 in SH-SY5Y cells.* Neurol Res, 2020. **42**(1): p. 47-54.

46. Huang, C., et al., *Inhibition of JAK1 by microRNA-708 promotes SH-SY5Y neuronal cell survival after oxygen and glucose deprivation and reoxygenation.* Neurosci Lett, 2018. **664**: p. 43-50.

47. Huang, L., Y. Shi, and L. Zhao, *Ginkgolide B Alleviates Learning and Memory Impairment in Rats With Vascular Dementia by Reducing Neuroinflammation via Regulating NF-kappaB Pathway.* Front Pharmacol, 2021. **12**: p. 676392.

48. Janssen, L., et al., *Inhibition of Fatty Acid Synthesis Aggravates Brain Injury, Reduces Blood-Brain Barrier Integrity and Impairs Neurological Recovery in a Murine Stroke Model.* Front Cell Neurosci, 2021. **15**: p. 733973.

49. Jia, Y., et al., *MiR-363-3p attenuates neonatal hypoxic-ischemia encephalopathy by targeting DUSP5.* Neurosci Res, 2021. **171**: p. 103-113.

50. Jiang, W.L., et al., *Tricin 7-glucoside protects against experimental cerebral ischemia by reduction of NF-kappaB and HMGB1 expression.* Eur J Pharm Sci, 2012. **45**(1-2): p. 50-7.

51. Jimenez-Almarza, A., et al., *Synthesis, neuroprotective and antioxidant capacity of PBN-related indanonitrones.* Bioorg Chem, 2019. **86**: p. 445-451.

52. Karuppagounder, S.S., et al., *In vitro ischemia suppresses hypoxic induction of hypoxia-inducible factor-1alpha by inhibition of synthesis and not enhanced degradation.* J Neurosci Res, 2013. **91**(8): p. 1066-75.

53. Lai, Z., et al., *Delta opioid peptide [d-Ala2, d-Leu5] enkephalin confers neuroprotection by activating delta opioid receptor-AMPK-autophagy axis against global ischemia.* Cell Biosci, 2020. **10**: p. 79.

54. Landgraf, A.D., et al., *Neuroprotective and Anti-neuroinflammatory Properties of Ebselen Derivatives and Their Potential to Inhibit Neurodegeneration.* ACS Chem Neurosci, 2020. **11**(19): p. 3008-3016.

55. Lee, O.H., et al., *Decreased expression of sirtuin 6 is associated with release of high mobility group box-1 after cerebral ischemia.* Biochem Biophys Res Commun, 2013. **438**(2): p. 388-94.

56. Li, J., et al., *Astilbin attenuates cerebral ischemia/reperfusion injury by inhibiting the TLR4/MyD88/NF-kappaB pathway.* Toxicol Res (Camb), 2019. **8**(6): p. 1002-1008.

57. Li, J. and L. Ma, *MiR-142-3p Attenuates Oxygen Glucose Deprivation/Reoxygenation-Induced Injury by Targeting FBXO3 in Human Neuroblastoma SH-SY5Y Cells.* World Neurosurg, 2020. **136**: p. e149-e157.

58. Li, T.F., et al., *Chrysin ameliorates cerebral ischemia/reperfusion (I/R) injury in rats by regulating the PI3K/Akt/mTOR pathway.* Neurochem Int, 2019. **129**: p. 104496.

59. Li, W.H., et al., *Baicalein attenuates caspase-independent cells death via inhibiting PARP-1 activation and AIF nuclear translocation in cerebral ischemia/reperfusion rats.* Apoptosis, 2020. **25**(5-6): p. 354-369.

60. Li, Y., et al., *miR-137 boosts the neuroprotective effect of endothelial progenitor cell-derived exosomes in oxyhemoglobin-treated SH-SY5Y cells partially via COX2/PGE2 pathway.* Stem Cell Res Ther, 2020. **11**(1): p. 330.

61. Lin, C.H., et al., *Neuroprotective effects of resveratrol against oxygen glucose deprivation induced mitochondrial dysfunction by activation of AMPK in SH-SY5Y cells with 3D gelatin scaffold.* Brain Res, 2020. **1726**: p. 146492.

62. Lin-Holderer, J., et al., *Fumaric acid esters promote neuronal survival upon ischemic stress through activation of the Nrf2 but not HIF-1 signaling pathway.* Neuropharmacology, 2016. **105**: p. 228-240.

63. Liu, J.Y., et al., *Cellular pharmacokinetics and pharmacodynamics mechanisms of ginkgo diterpene lactone and its modulation of P-glycoprotein expression in human SH-SY5Y cells.* Biomed Chromatogr, 2019. **33**(12): p. e4692.

64. Liu, N., et al., *LncRNA AC136007.2 alleviates cerebral ischemic-reperfusion injury by suppressing autophagy.* Aging (Albany NY), 2021. **13**(15): p. 19587-19597.

65. Liu, Q., et al., *Antioxidant effects of ginkgolides and bilobalide against cerebral ischemia injury by activating the Akt/Nrf2 pathway in vitro and in vivo.* Cell Stress Chaperones, 2019. **24**(2): p. 441-452.

66. Liu, W.F., G.-S.; Ou, Y,; Xu, J.; Zhang, Z.-J.; Zhang, G.-X.; Sun, Y.-W.; Li, S.; Jian, J., *Neuroprotective effect of apocynin nitrone in oxygen glucose deprivation-treated SH-SY5Y cells and rats with ischemic stroke.* Tropical Journal of Pharmaceutical Research, 2016. **15**(8): p. 1681-1689.

67. Liu, Y., et al., *Human Ischaemic Cascade Studies Using SH-SY5Y Cells: a Systematic Review and Meta-Analysis.* Transl Stroke Res, 2018. **9**(6): p. 564-574.

68. Liu, Y., et al., *Tissue kallikrein protects SH-SY5Y neuronal cells against oxygen and glucose deprivation-induced injury through bradykinin B2 receptor-dependent regulation of autophagy induction.* J Neurochem, 2016. **139**(2): p. 208-220.

69. Liu, Z.D., et al., *MicroRNA-130b inhibits cerebral ischemia/reperfusion induced cell apoptosis via regulation of IRF1.* Eur Rev Med Pharmacol Sci, 2020. **24**(23): p. 12334-12341.

70. Lu, T., et al., *The mechanism on phosphorylation of Hsp20Ser16 inhibit GA stress and ER stress during OGD/R.* PLoS One, 2019. **14**(3): p. e0213410.

71. Luan, H., et al., *Rosmarinic acid protects against experimental diabetes with cerebral ischemia: relation to inflammation response.* J Neuroinflammation, 2013. **10**: p. 28.

72. Marmol, F., J. Sanchez, and A. Martinez-Pinteno, *Effects of uric acid on oxidative and nitrosative stress and other related parameters in SH-SY5Y human neuroblastoma cells.* Prostaglandins Leukot Essent Fatty Acids, 2021. **165**: p. 102237.

73. Marutani, E., et al., *A novel hydrogen sulfide-releasing N-methyl-D-aspartate receptor antagonist prevents ischemic neuronal death.* J Biol Chem, 2012. **287**(38): p. 32124-35.

74. McCune, C.D., et al., *"Zipped Synthesis" by Cross-Metathesis Provides a Cystathionine beta-Synthase Inhibitor that Attenuates Cellular H2S Levels and Reduces Neuronal Infarction in a Rat Ischemic Stroke Model.* ACS Cent Sci, 2016. **2**(4): p. 242-52.

75. Meng, Q., P. Yang, and Y. Lu, *MicroRNA-410 serves as a candidate biomarker in hypoxic-ischemic encephalopathy newborns and provides neuroprotection in oxygen-glucose deprivation-injured PC12 and SH-SY5Y cells.* Brain Behav, 2021. **11**(8): p. e2293.

76. Miners, S., et al., *Reduced vascular endothelial growth factor and capillary density in the occipital cortex in dementia with Lewy bodies.* Brain Pathol, 2014. **24**(4): p. 334-43.

77. Nampoothiri, S.S. and G.K. Rajanikant, *miR-9 Upregulation Integrates Post-ischemic Neuronal Survival and Regeneration In Vitro.* Cell Mol Neurobiol, 2019. **39**(2): p. 223-240.

78. Niu, G., et al., *Role of Hypoxia-Inducible Factors 1alpha (HIF1alpha) in SH-SY5Y Cell Autophagy Induced by Oxygen-Glucose Deprivation.* Med Sci Monit, 2018. **24**: p. 2758-2766.

79. Park, S.Y., et al., *Kalopanacis Cortex extract-capped gold nanoparticles activate NRF2 signaling and ameliorate damage in human neuronal SH-SY5Y cells exposed to oxygen-glucose deprivation and reoxygenation.* Int J Nanomedicine, 2017. **12**: p. 4563-4578.

80. Ping, F., et al., *Cx32 inhibits the autophagic effect of Nur77 in SH-SY5Y cells and rat brain with ischemic stroke.* Aging (Albany NY), 2021. **13**(18): p. 22188-22207.

81. Roy, K., D. Maji, and I. Deb, *Increase of Cry 1 expression is a common phenomenon of the disturbed circadian clock in ischemic stroke and opioid addiction.* Biochem Biophys Res Commun, 2021. **558**: p. 8-13.

82. Ruan, L., et al., *Activation of Adenosine A1 Receptor in Ischemic Stroke: Neuroprotection by Tetrahydroxy Stilbene Glycoside as an Agonist.* Antioxidants (Basel), 2021. **10**(7).

83. Ruan, Z.F., et al., *MiR-370 accelerated cerebral ischemia reperfusion injury via targeting SIRT6 and regulating Nrf2/ARE signal pathway.* Kaohsiung J Med Sci, 2020. **36**(9): p. 741-749.

84. Shan, W., et al., *Upregulation of miR-499a-5p Decreases Cerebral Ischemia/Reperfusion Injury by Targeting PDCD4.* Cell Mol Neurobiol, 2022. **42**(7): p. 2157-2170.

85. Shi, R., et al., *Excessive autophagy contributes to neuron death in cerebral ischemia.* CNS Neurosci Ther, 2012. **18**(3): p. 250-60.

86. Shi, X., et al., *Panax notoginseng saponins provide neuroprotection by regulating NgR1/RhoA/ROCK2 pathway expression, in vitro and in vivo.* J Ethnopharmacol, 2016. **190**: p. 301-12.

87. Shi, Y., et al., *MiR-155-5p accelerates cerebral ischemia-reperfusion injury via targeting DUSP14 by regulating NF-kappaB and MAPKs signaling pathways.* Eur Rev Med Pharmacol Sci, 2020. **24**(3): p. 1408-1419.

88. Shi, Y., et al., *MicroRNA-532-5p protects against cerebral ischemia-reperfusion injury by directly targeting CXCL1.* Aging (Albany NY), 2021. **13**(8): p. 11528-11541.

89. Shi, Y.S., et al., *Nomilin protects against cerebral ischemia-reperfusion induced neurological deficits and blood-brain barrier disruption via the Nrf2 pathway.* Food Funct, 2019. **10**(9): p. 5323-5332.

90. Shin, T.H., et al., *Restoration of Polyamine Metabolic Patterns in In Vivo and In Vitro Model of Ischemic Stroke following Human Mesenchymal Stem Cell Treatment.* Stem Cells Int, 2016. **2016**: p. 4612531.

91. Sinoy, S., et al., *Amikacin Inhibits miR-497 Maturation and Exerts Post-ischemic Neuroprotection.* Mol Neurobiol, 2017. **54**(5): p. 3683-3694.

92. Song, J., et al., *Inhibition of FOXO3a/BIM signaling pathway contributes to the protective effect of salvianolic acid A against cerebral ischemia/reperfusion injury.* Acta Pharm Sin B, 2019. **9**(3): p. 505-515.

93. Tajes, M., et al., *Nitro-oxidative stress after neuronal ischemia induces protein nitrotyrosination and cell death.* Oxid Med Cell Longev, 2013. **2013**: p. 826143.

94. Tan, Y., et al., *MicroRNA-126a-5p Exerts Neuroprotective Effects on Ischemic Stroke via Targeting NADPH Oxidase 2.* Neuropsychiatr Dis Treat, 2021. **17**: p. 2089-2103.

95. Tang, S.C., et al., *Evidence for a detrimental role of TLR8 in ischemic stroke.* Exp Neurol, 2013. **250**: p. 341-7.

96. Tian, C., et al., *lncRNA NR_120420 promotes SH-SY5Y cells apoptosis by regulating NF-kappaB after oxygen and glucose deprivation.* Gene, 2020. **728**: p. 144285.

97. Wang, D., et al., *Apelin receptor homodimer inhibits apoptosis in vascular dementia.* Exp Cell Res, 2021. **407**(1): p. 112739.

98. Wang, D.W., et al., *LncRNA SNHG1 protects SH-SY5Y cells from hypoxic injury through miR-140-5p/Bcl-XL axis.* Int J Neurosci, 2021. **131**(4): p. 336-345.

99. Wang, H., et al., *Long Non-coding RNA TUG1 Sponges Mir-145a-5p to Regulate Microglial Polarization After Oxygen-Glucose Deprivation.* Front Mol Neurosci, 2019. **12**: p. 215.

100. Wang, H.F., et al., *Endoplasmic reticulum stress regulates oxygen-glucose deprivation-induced parthanatos in human SH-SY5Y cells via improvement of intracellular ROS.* CNS Neurosci Ther, 2018. **24**(1): p. 29-38.

101. Wang, J., et al., *Long Non-coding RNA H19 Induces Cerebral Ischemia Reperfusion Injury via Activation of Autophagy.* Aging Dis, 2017. **8**(1): p. 71-84.

102. Wang, J., et al., *Trametenolic acid B protects against cerebral ischemia and reperfusion injury through modulation of microRNA-10a and PI3K/Akt/mTOR signaling pathways.* Biomed Pharmacother, 2019. **112**: p. 108692.

103. Wang, M., et al., *LncRNA NKILA upregulation mediates oxygen glucose deprivation/re-oxygenation-induced neuronal cell death by inhibiting NF-kappaB signaling.* Biochem Biophys Res Commun, 2018. **503**(4): p. 2524-2530.

104. Wang, N., et al., *The protective activity of imperatorin in cultured neural cells exposed to hypoxia re-oxygenation injury via anti-apoptosis.* Fitoterapia, 2013. **90**: p. 38-43.

105. Wang, R., et al., *miR-186-5p Promotes Apoptosis by Targeting IGF-1 in SH-SY5Y OGD/R Model.* Int J Biol Sci, 2018. **14**(13): p. 1791-1799.

106. Wang, T., et al., *Picroside II Protects SH-SY5Y Cells From Autophagy and Apoptosis Following Oxygen Glucose Deprivation/Reoxygen Injury by Inhibiting JNK Signal Pathway.* Anat Rec (Hoboken), 2019. **302**(12): p. 2245-2254.

107. Wang, Y. and M. Xu, *miR-380-5p facilitates NRF2 and attenuates cerebral ischemia/reperfusion injury-induced neuronal cell death by directly targeting BACH1.* Transl Neurosci, 2021. **12**(1): p. 210-217.

108. Wang, Z.Q., et al., *MicroRNA Let-7i Is a Promising Serum Biomarker for Post-stroke Cognitive Impairment and Alleviated OGD-Induced Cell Damage in vitro by Regulating Bcl-2.* Front Neurosci, 2020. **14**: p. 215.

109. Wen, Y., et al., *Suppression of lncRNA SNHG15 protects against cerebral ischemia-reperfusion injury by targeting miR-183-5p/FOXO1 axis.* Am J Transl Res, 2020. **12**(10): p. 6250-6263.

110. Wu, L., et al., *Curcumin exerts protective effects against hypoxiareoxygenation injury via the enhancement of apurinic/apyrimidinic endonuclease 1 in SHSY5Y cells: Involvement of the PI3K/AKT pathway.* Int J Mol Med, 2020. **45**(4): p. 993-1004.

111. Wu, S., et al., *In Vitro Evaluation of the Neuroprotective Effect of Panax notoginseng Saponins by Activating the EGFR/PI3K/AKT Pathway.* Evid Based Complement Alternat Med, 2020. **2020**: p. 1403572.

112. Xing, C., G. Yan, and Q. Liu, *Inhibition of GPR4 attenuates SH-SY5Y cell injury in cerebral ischemia/reperfusion via anti-apoptotic pathways.* Acta Biochim Pol, 2021. **68**(2): p. 181-186.

113. Xu, Z., W. Liu, and H. Huang, *Astragaloside IV Alleviates Cerebral Ischemia-Reperfusion Injury by Activating the Janus Kinase 2 and Signal Transducer and Activator of Transcription 3 Signaling Pathway.* Pharmacology, 2020. **105**(3-4): p. 181-189.

114. Yan, X.L., et al., *S-Nitrosylation of proline-rich tyrosine kinase 2 involves its activation induced by oxygen-glucose deprivation.* Neurosci Lett, 2015. **597**: p. 90-6.

115. Yan, Y., et al., *SNHG12 inhibits oxygenglucose deprivationinduced neuronal apoptosis via the miR181a5p/NEGR1 axis.* Mol Med Rep, 2020. **22**(5): p. 3886-3894.

116. Yang, H., et al., *Mir-184 Contributes to Brain Injury Through Targeting PPAP2B Following Ischemic Stroke in Male Rats.* Front Mol Neurosci, 2021. **14**: p. 613887.

117. Yang, T., et al., *Piperlonguminine is neuroprotective in experimental rat stroke.* Int Immunopharmacol, 2014. **23**(2): p. 447-51.

118. Yang, T., et al., *Punicalin Alleviates OGD/R-Triggered Cell Injury via TGF-beta-Mediated Oxidative Stress and Cell Cycle in Neuroblastoma Cells SH-SY5Y.* Evid Based Complement Alternat Med, 2021. **2021**: p. 6671282.

119. Yao, X., et al., *Upregulation of miR-496 decreases cerebral ischemia/reperfusion injury by negatively regulating BCL2L14.* Neurosci Lett, 2019. **696**: p. 197-205.

120. Yi, Z., et al., *Overexpression of miR-217-5p protects against oxygen-glucose deprivation/reperfusion-induced neuronal injury via inhibition of PTEN.* Hum Cell, 2020. **33**(4): p. 1026-1035.

121. Yin, M., et al., *LncRNA TUG1 Demethylated by TET2 Promotes NLRP3 Expression, Contributes to Cerebral Ischemia/Reperfusion Inflammatory Injury.* ASN Neuro, 2021. **13**: p. 17590914211003247.

122. Zappala, A., et al., *Neuroprotective effects of Rosmarinus officinalis L. extract in oxygen glucose deprivation (OGD)-injured human neural-like cells.* Nat Prod Res, 2021. **35**(4): p. 669-675.

123. Zhang, D., et al., *Procaspase-9 induces its cleavage by transnitrosylating XIAP via the Thioredoxin system during cerebral ischemia-reperfusion in rats.* Sci Rep, 2016. **6**: p. 24203.

124. Zhang, H., et al., *Upregulation of miR-376c-3p alleviates oxygen-glucose deprivation-induced cell injury by targeting ING5.* Cell Mol Biol Lett, 2019. **24**: p. 67.

125. Zhang, J.F., et al., *MicroRNA-25 Negatively Regulates Cerebral Ischemia/Reperfusion Injury-Induced Cell Apoptosis Through Fas/FasL Pathway.* J Mol Neurosci, 2016. **58**(4): p. 507-16.

126. Zhang, L., et al., *miR-155 Knockdown Protects against Cerebral Ischemia and Reperfusion Injury by Targeting MafB.* Biomed Res Int, 2020. **2020**: p. 6458204.

127. Zhang, Y.T., et al., *(Z)-ligustilide increases ferroportin1 expression and ferritin content in ischemic SH-SY5Y cells.* Eur J Pharmacol, 2016. **792**: p. 48-53.

128. Zhang, Z.B., et al., *miRNA-7a-2-3p Inhibits Neuronal Apoptosis in Oxygen-Glucose Deprivation (OGD) Model.* Front Neurosci, 2019. **13**: p. 16.

129. Zhang, Z.H., et al., *Circ-camk4 involved in cerebral ischemia/reperfusion induced neuronal injury.* Sci Rep, 2020. **10**(1): p. 7012.

130. Zhao, J. and B. Wang, *MiR-7-5p Enhances Cerebral Ischemia-Reperfusion Injury by Degrading sirt1 mRNA.* J Cardiovasc Pharmacol, 2020. **76**(2): p. 227-236.

131. Zhao, L.P., et al., *Oxygen glucose deprivation (OGD)/re-oxygenation-induced in vitro neuronal cell death involves mitochondrial cyclophilin-D/P53 signaling axis.* Neurochem Res, 2013. **38**(4): p. 705-13.

132. Zhi, S.M., et al., *Melatonin reduces OGD/R-induced neuron injury by regulating redox/inflammation/apoptosis signaling.* Eur Rev Med Pharmacol Sci, 2020. **24**(3): p. 1524-1536.

133. Zhou, Z., et al., *Suppression of lncRNA RMRP ameliorates oxygen-glucose deprivation/re-oxygenation-induced neural cells injury by inhibiting autophagy and PI3K/Akt/mTOR-mediated apoptosis.* Biosci Rep, 2019. **39**(6).

134. Zuo, M.L., et al., *miR-652 protects rats from cerebral ischemia/reperfusion oxidative stress injury by directly targeting NOX2.* Biomed Pharmacother, 2020. **124**: p. 109860.

135. Ingrassia, R., et al., *1B/(-)IRE DMT1 expression during brain ischemia contributes to cell death mediated by NF-kappaB/RelA acetylation at Lys310.* PLoS One, 2012. **7**(5): p. e38019.

136. Wang, J., et al., *KIF2 mediates the neuroprotection in cerebral ischaemia injury by affecting NF-kappaB pathway.* Clin Exp Pharmacol Physiol, 2020. **47**(2): p. 274-280.

137. Jin, X., et al., *MicroRNA-19a mediates neuroprotection through the PTEN/AKT pathway in SK-N-SH cells after oxygen-glucose deprivation/reoxygenation injury.* Gen Physiol Biophys, 2020. **39**(3): p. 259-268.

138. Lehane, C., et al., *Carbimazole is an inhibitor of protein synthesis and protects from neuronal hypoxic damage in vitro.* J Pharmacol Exp Ther, 2013. **347**(3): p. 781-93.

139. Li, S.H., et al., *Decreased miR-146a expression in acute ischemic stroke directly targets the Fbxl10 mRNA and is involved in modulating apoptosis.* Neurochem Int, 2017. **107**: p. 156-167.

140. Olechnowicz, S.W., A.O. Fedele, and D.J. Peet, *Hypoxic induction of the regulator of G-protein signalling 4 gene is mediated by the hypoxia-inducible factor pathway.* PLoS One, 2012. **7**(9): p. e44564.

141. Rosenthal, L.M., et al., *Neuroprotection via RNA-binding protein RBM3 expression is regulated by hypothermia but not by hypoxia in human SK-N-SH neurons.* Hypoxia (Auckl), 2017. **5**: p. 33-43.

142. Soh, H., M. Wasa, and M. Fukuzawa, *Hypoxia upregulates amino acid transport in a human neuroblastoma cell line.* J Pediatr Surg, 2007. **42**(4): p. 608-12.

143. Yanagita, T., et al., *Possible involvement of the expression and phosphorylation of N-Myc in the induction of HMGA1a by hypoxia in the human neuroblastoma cell line.* Neurosci Lett, 2005. **374**(1): p. 47-52.

144. Zhou, X., et al., *MicroRNA-146a down-regulation correlates with neuroprotection and targets pro-apoptotic genes in cerebral ischemic injury in vitro.* Brain Res, 2016. **1648**(Pt A): p. 136-143.

145. Pedersen, E.D., et al., *CD59 efficiently protects human NT2-N neurons against complement-mediated damage.* Scand J Immunol, 2007. **66**(2-3): p. 345-51.

146. Pedersen, E.D., et al., *Expression of complement regulators and receptors on human NT2-N neurons--effect of hypoxia and reoxygenation.* Mol Immunol, 2007. **44**(9): p. 2459-68.

147. Pires Monteiro, S., et al., *Neuroprotective effect of hypoxic preconditioning and neuronal activation in a human model of the ischemic penumbra.* J Neural Eng, 2021.
